# NrdR Transcription Regulation: Global Proteome Analysis and Its Role in *Escherichia coli* Viability and Virulence

**DOI:** 10.1371/journal.pone.0157165

**Published:** 2016-06-08

**Authors:** Vankadari Naveen, Chwan-Deng Hsiao

**Affiliations:** 1 Molecular Cell Biology, Taiwan International Graduate Program, Graduate Institute of Life Sciences, National Defense Medical Center and Academia Sinica, Taipei, Taiwan; 2 Institute of Molecular Biology, Academia Sinica, Taipei, 115, Taiwan; University of Cape Town, SOUTH AFRICA

## Abstract

Bacterial ribonucleotide reductases (RNRs) play an important role in the synthesis of dNTPs and their expression is regulated by the transcription factors, NrdR and Fur. Recent transcriptomic studies using deletion mutants have indicated a role for NrdR in bacterial chemotaxis and in the maintenance of topoisomerase levels. However, NrdR deletion alone has no effect on bacterial growth or virulence in infected flies or in human blood cells. Furthermore, transcriptomic studies are limited to the deletion strain alone, and so are inadequate for drawing biological implications when the NrdR repressor is active or abundant. Therefore, further examination is warranted of changes in the cellular proteome in response to both NrdR overexpression, as well as deletion, to better understand its functional relevance as a bacterial transcription repressor. Here, we profile bacterial fate under conditions of overexpression and deletion of NrdR in *E*. *coli*. Biochemical assays show auxiliary zinc enhances the DNA binding activity of NrdR. We also demonstrate at the physiological level that increased *nrdR* expression causes a significant reduction in bacterial growth and fitness even at normal temperatures, and causes lethality at elevated temperatures. Corroborating these direct effects, global proteome analysis following NrdR overexpression showed a significant decrease in global protein expression. In parallel, studies on complementary expression of downregulated essential genes *polA*, *eno* and *thiL* showed partial rescue of the fitness defect caused by NrdR overexpression. Deletion of downregulated non-essential genes *ygfK* and *trxA* upon NrdR overexpression resulted in diminished bacterial growth and fitness suggesting an additional role for NrdR in regulating other genes. Moreover, in comparison with NrdR deletion, *E*. *coli* cells overexpressing NrdR showed significantly diminished adherence to human epithelial cells, reflecting decreased bacterial virulence. These results suggest that elevated expression of NrdR could be a suitable means to retard bacterial growth and virulence, as its elevated expression reduces bacterial fitness and impairs host cell adhesion.

## Introduction

For all organisms, timely and temporal regulation of gene expression and its translation to protein level is crucial for cell proliferation. These complex multistep events are controlled by various metabolic processes and their inter-regulation. Genes involved in ATP and DNA biogenesis play a key role in DNA duplication and cell division [1]. Ribonucleotide reductases (RNRs) represent one such essential class of enzymes that catalyses the conversion of all four ribonucleotides (rNTPs) into their corresponding 2’-deoxyribonucleotides (dNTPs), providing the precursors for DNA synthesis and repair [2, 3]. Bacterial RNRs are grouped into three classes: class I, comprising Ia and Ic (*nrdAB*) and Ib (*nrdHIEF*); class II (*nrdJ*); and class III (*nrdDG*). Although class 1a and 1c are both regulated by *nrdAB* genes, class 1c RNRs can be distinguished from class 1a RNRs by the protein radical that is generated through an Mn^4^-O-Fe^3^ center and are found in species like *Chlamydia trachomatis* [1, 4]. The three classes of RNRs differ in their primary structure and cofactor requirements but share a relatively similar catalytic mechanism [3, 5]. However, the distribution and regulation patterns of RNRs differ among bacterial species and, even among subtypes, they are not well conserved [6, 7]. Most enterobacterial species like *E*. *coli*, *Helicobactor* and *Klebsiella* encode the two major classes of RNRs (Iab and III), but a few bacterial species such as *Pseudomonas* encode class II and lack RNRs of class Ib, which is a large known group [8, 9].

The proteins, NrdR and Fur, have been described as novel transcription repressors and have been shown to regulate the expression of various RNRs [10–14]. However, Fur predominantly regulates the expression of a large group class Ib or *nrdHIEF*, whereas NrdR acts as a repressor for all three classes of RNRs in bacteria. *E*. *coli* NrdR is composed of an N-terminal non-classical zinc-binding region and a unique C-terminal ATP-cone domain [1], which classifies NrdR within the ATP-cone family of proteins. Though the ATP-cone is a unique feature of NrdR, mutations in this domain only cause weak DNA binding but do not abolish its activity entirely [13, 14]. In contrast, the non-classical zinc-binding N-terminal region of NrdR has been found to be crucial for DNA binding activity in *Streptomyces* species [14]. Interestingly, the presence of ATP, dATP or ADP has been found to inhibit NrdR DNA binding activity, and it has been demonstrated that either the apo form or AMP/dAMP is preferred by NrdR for binding to its target DNA substrate [11]. Though it is unclear that how nucleotide exchange takes place in NrdR, the nucleotide exchange process is known to play a key role in regulating the conformation or oligomeric state of NrdR [1, 11]. Furthermore, NrdR shows a preference for binding to several promoter regions linked to so-called NrdR boxes or NrdR promoter-binding regions across the genome [15, 16] and to the cognate DNA substrates either containing *nrdA* or *ribX* promoter regions [11, 13].

The transcription repressor NrdR is usually found clustered with RNR genes or with genes that are involved in primosome assembly and bacterial DNA replication, such as *dnaA*, *dnaB*, *dnaI* and *polA*, in different bacterial species [15, 17, 18]. In addition to their regulatory role in the expression of RNRs, both transcription repressors NrdR and Fur have been found to interact with Thioredoxin (TrxA), which plays a key role in regulating redox regulatory pathways and signal transduction in bacteria [19]. NrdR has also been shown to associate with several other bacterial proteins such as RibD, RibE, GlyS, ThiL, YdbK, Frc and YfhM [20–22], via unknown mechanisms. This suggests that cellular expression of NrdR and regulation of its levels have biological relevance for the maintenance of bacterial homeostasis and for other important biological functions in addition to RNR regulation. Recent transcriptomic studies in *E*. *coli* under NrdR deletion alone have shown an increase in the mRNA expression levels of a few RNRs and a decrease in the genes corresponding to motility and chemotaxis [8, 9]. However, in contrast, transcriptomic studies under NrdR deletion alone in *Pseudomonas* have shown upregulation of RNRs, but no significant decrease in levels of other genes [9]. These two latter studies demonstrate the variability in NrdR regulation in different bacterial species. In addition NrdR deletion alone showed no effect on bacterial growth profiles, which could be possibly due to the effective role contributed by Fur in regulating class 1b large group of RNRs. Furthermore, although some variability in the degree of bacterial adherence to host cells has been shown, deletion of NrdR alone either in *P*. *aeruginosa* or *S*. *pyogenes* revealed no difference in host infectivity in *Drosophila* and mammalian blood cell models, respectively [8, 9, 23]. Thus it would be interesting to determine bacterial fate and growth profiles during active NrdR transcription repression using overexpression studies in bacteria. It has been revealed that NrdR abundance increases naturally (~2.5-fold under a log2 scale) during the bacterial stationery growth phase and under conditions of mRNA abundance, tryptophan supplementation and oxidative stress [24–26]. Thus, together, the aforementioned non-RNR regulatory functions of NrdR and its drastic upregulation under several physiological conditions suggests multiple roles for NrdR in bacterial homeostasis, with this latter being regulated by changes in NrdR expression levels.

As previous studies have been limited to mRNA expression alone following NrdR deletion, which are inadequate for drawing biological conclusions for situations when the repressor is active or upregulated, further studies at the protein level can provide greater insights into the role of NrdR in bacterial fitness. This information could clarify the physiological basis of bacterial fitness and virulence when transcription repressors like NrdR are active or abundant in the cell. Here, we provide a comprehensive global proteome analysis under conditions of NrdR overexpression and deletion, with cognate fitness rescue or deterioration studies, which reveal potential alternative targets of NrdR in regulating bacterial growth and fitness relating to bacterial virulence. Furthermore, physiological studies suggest that elevated levels of NrdR at early stages retards bacterial growth and fitness.

## Materials and Methods

### Cloning, protein expression and purification

The full length ORF of NrdR from BW25113 [27] was cloned between the *Nde*I and *Xho*I restriction sites of the pET21b expression vector with a C-terminal His tag and expressed in *E*. *coli* BL21 (DE3). We also observed the complete sequence similarity between the variants of *E*. *coli*, both pathogenic (LF82 and O157:H7) and non-pathogenic (MG1655 and ME5305). Cells were cultured in Luria-Bertani (LB) broth containing 100 μg/ml of ampicillin at 37°C and induced with 1 mM isopropyl-thio-D-galactopyranoside (IPTG) at 0.45 OD. All purification procedures were performed at 4°C. The cell pellets were pooled and then suspended in 10 ml of 20 mM Tris-HCl (pH 7.8), 500 mM NaCl, and 10 mM imidazole (buffer A) per gram of cell paste. Samples were passed through an M-110L microfluidizer apparatus (Microfluidics), and then centrifuged at 16000 RPM for 30 min. The supernatant was filtered and loaded onto a HisTrap Ni^+2^-chelating column (GE Healthcare) equilibrated with buffer A. The column was washed with 50 ml of buffer A, and bound NrdR was eluted with a linear gradient of 20–500 mM imidazole in buffer A. Fractions containing NrdR were pooled and dialyzed against 20 mM Tris-HCl (pH 8.4), 100 mM NaCl, 2 mM MgCl_2_ and 10 mM β-mercaptoethanol (buffer B), and applied to size exclusion chromatography through a HiLoad 16/600 Superdex 200-pg column (GE Healthcare) equilibrated with buffer B. NrdR purity was >98%, as assessed by SDS-PAGE and confirmed by MALDI-MS. To overcome the oligomerization of NrdR, we applied a previously described protocol [11] with some modifications. First, we unfolded the Ni-column-eluted protein with 8M urea in a buffer containing 20 mM Tris-HCl (pH 8.4), 400 mM NaCl, 10 mM β-mercaptoethanol and then allowed refolding through a stepwise reduction of urea concentrations (6M, 4M, 2M, 0M) by dialysis. Finally, protein was dialyzed against urea-free buffer twice and then purified by gel filtration as described above, thereby obtaining the “apo-form” of refolded protein.

### Gel retardation assay

The DNA substrate of the *E*. *coli nrdAB* promoter region was prepared by PCR amplification as previously described [11] without biotin labeling. Binding assays were carried out in a final volume of 20 μl reaction mixture containing 20 mM Tris-Cl (pH 9.5), 100 mM KCl, 2 mM MgCl_2_, 5% glycerol, and 5 mM BME with 250 nM DNA substrate. Individual reaction mixtures were incubated at 27°C for 30 min with increasing concentrations (5, 10, 15 and 25 μM) of purified NrdR, and then assayed in the presence or absence of an additional 3 μM ZnSO_4_. To further determine the importance of zinc for DNA binding, 25 μM of NrdR was incubated with 250 nM of DNA substrate at 27°C for 30 min with increasing concentrations of zinc (0.1 μM to 6 μM). The protein-DNA complexes were electrophoresed on a 1.2% agarose gel in TAE buffer. Electrophoresed DNA was stained with EtBr and imaged using the Avegene^™^ system.

### Bacterial synchronous growth assay

Bacterial strains of wild-type *E*. *coli* BW25113, NrdR-deletion (ΔNrdR) [27, 28] containing empty vector control, and overexpression of NrdR in BW25113 (OE-NrdR) were grown in LB medium containing 100 μg/ml ampicillin. Log phase *E*. *coli* cultures (0.45 OD) were serially diluted in ampicillin-containing LB medium to achieve ~0.1 OD, and the corresponding individually-diluted cultures were seeded in sterile ELISA (96 well) plates containing 100 μl LB medium, 100 μg/ml ampicillin and 0.5 mM IPTG. The plates incubated at 37°C and 160 RPM for synchronous growth for 18 h and monitored by Infinity 200 Pro (Tecan Ltd.), while OD at 595 nM was recorded every 15 min. The measured bacterial growth was plotted as time in hours vs. OD at 595 nM from the recorded raw values using Magellan software (Tecan Ltd.). To monitor the indicated bacterial growth in a simulated host cell environment, the same procedure was followed except that bacterial cells were grown in mammalian cell culture Dulbecco’s Modified Eagle’s Medium (DMEM).

In order to assess the specific effects of NrdR overexpression and to have a negative expression control, both WT and ΔNrdR *E*. *coli* strains were subjected to overexpression studies with a neutral protein β-galactosidase from LacZ operon [29], and the corresponding strains were grown as described above for the WT and ΔNrdR *E*. *coli* strains carrying empty vector and OE-NrdR. The respective WT and ΔNrdR *E*. *coli* strains carrying the β-galactosidase overexpression plasmid also underwent a growth fitness assay (spot assay and streak plate) on LB agar containing 100 μg/ml ampicillin, 0.5 mM IPTG and 40 μg/ml X-gal and incubated at 37°C. For the spot assay, the mid log phase cultures of above grown in antibiotic medium were serially-diluted and the respective dilutions were spotted onto the above-described LB agar plates and incubated over-night along with the OE-NrdR strain. For the streak plate assay, 5 μl of the same mid log phase cultures were streaked onto the above-described LB medium and incubated overnight and assessed for bacterial growth.

To further corroborate the individual growth curves of WT, ΔNrdR and OE-NrdR *E*. *coli* strains alone, CFU analysis was performed as described previously [30, 31]. In brief, 1 mL aliquots of individual *E*. *coli* cultures were collected for every two hours and subjected to serial 10-fold dilutions in sterile LB broth, and dilutions were then spread on LB agar plates containing 100 μg/ml ampicillin to determine CFU/ml using the formula [CFU/ml = (no. of colonies x dilution factor) / volume spread on culture plate].

### Bacterial fitness assay

The WT *E*. *coli* BW25113, ΔNrdR and OE-NrdR strains were subjected to an *in vivo* viability or fitness test by adopting the spot assay as described previously [32]. The *E*. *coli* host carrying an empty vector of pET21b was used as a negative control, and *E*. *coli* lacking DnaK (Hsp70) (ΔDnaK) was used as a positive reference control. DnaK is essential for bacterial viability only under heat shock conditions, and its deletion under elevated temperatures causes lethality [32]. To elucidate the key regions of NrdR for fitness recovery, the NrdR Arg patch mutant (Arg 26–29 to Ala), N-terminal- and ATP cone domain-truncated proteins were complementarily overexpressed in an NrdR deletion background to assess bacterial growth. The single colony transformants were selected at 30°C onto LB agar plates with respective antibiotic selection. Fresh overnight cultures were grown from the individual single colonies, and the *A*600 of individually-grown cultures were adjusted to 0.2 OD by the addition of LB medium. Serial dilutions (10-fold) of these cultures were spotted onto antibiotic-free agar plates containing LB and 0.5 mM IPTG and then incubated for 10 hours at 37°C or 42°C to evaluate bacterial fitness in response to deletion or overexpression of NrdR.

### Preparation of *E*. *coli* cell extract for global protein profiling

All the bacterial cultures were grown as described above for the synchronous growth assays. In brief, WT *E*. *coli* BW25113 (do not harbor any native plasmid), ΔNrdR and OE-NrdR *E*. *coli* strains were grown with agitation at 37°C in LB media containing 0.5 mM IPTG. At mid-exponential phase (OD_600_ of 0.45), the cells were harvested by centrifugation at 5,000 rpm for 10 min at 4°C. As the OE-NrdR strain under the IPTG induction grew slowly, we incubated for nearly seven hours to obtain cells of the desired OD. The fresh cell pellet was then subjected to crude cell extract preparation as previously described [33]. In brief, the cells were suspended in modified ice-cold breakage buffer (300 mM NaCl, 5 mM DTT, 5 mM MgCl_2_, 1 mM PMSF, 10% glycerol and 50 mM Tris-HCl, pH 8.0). To digest nucleic acids, 20 units of DNase I (Sigma) and 20 units of RNase (Ambion) were added, and samples were incubated for 15 min on ice. Protease inhibitor cocktail (Thermo Scientific) at a final concentration of 1 mM was added to the individual cell extract, and then subjected to three rounds of quick freeze-thaw cycles and further lysis by sonification at 20% amplitude with an interval of 1 sec on/3sec off using a Bronson sonifier. After complete lysis, unbroken cells were removed by centrifugation at 1000 rpm for 10 min at 4°C. The supernatant of individual protein concentrations was determined to be approximately 7 mg/ml of the total cell lysate by Bradford assay, and protein samples were then subjected to further enzymatic digestion.

### Tryptic digestion and TMT labeling

Enzymatic digestions of 20 μl of *E*. *coli* cell lysates from WT, ΔNrdR and OE-NrdR strains (approximately 7 mg/ml) were performed as described in the standard protocol (Thermo Scientific, TMT mass tagging and sample preparation) [34]. In brief, individual samples were suspended in 20 μl reduction buffer (200 mM DTE, 8 M urea and 25 mM ammonium bicarbonate pH 8.5) and incubated at 37°C for 1 h. Samples were further reduced with buffer containing 25 mM ammonium bicarbonate pH 8.5 and 20 mM iodoacetamide (IAM) in the dark at room temperature for 1 h. The reduced samples were digested first with Lys-C protease for 4 h and then with trypsin for 16 h at a ratio of 1 U for 50 μg of protein sample. The reaction was quenched by adding 1% formic acid to a final concentration of 0.1%. The individual samples were dried in a Speed-Vac and dissolved in 10 μl of 0.1% formic acid. The acidified samples were purified using pre-equilibrated C_18_ Zip-Tip columns (Merck Millipore), and bound peptides were eluted in 30 μl of buffer containing 50% acetonitrile and 0.1% formic acid. The individual samples were dried by Speed-Vac, and final concentrations of approximately 5 μg/ml from each sample was dissolved in 100 μl of 100 mM TEAB (triethyl ammonium bicarbonate), i.e. an equilibration buffer for TMT (Tandom Mass Tagging) labeling (Thermo). The three mass tags of TMT 126, 127 and 128 from the SixPlex kit (TMT^6^) were labeled for WT, ΔNrdR and OE-NrdR peptides, respectively, at a ratio of 1:1.7. The individually-labeled samples were incubated at 37°C for 1 h, and the reaction was quenched by adding 5% hydroxylamine and incubating for 20 min to ensure complete quenching and to abolish further reaction. The individually-labeled peptide samples were mixed and purified using C_18_ Zip-Tip columns as described previously. The eluted samples were dried and suspended in 5% (v/v) acetonitrile/1% (v/v) formic acid in water prior to LC-MS/MS analysis.

### LC-MS/MS analysis

Quantitative proteomics (*Shotgun proteomic identifications of peptides*) was conducted through NanoLC−nanoESI-MS/MS analysis on a nanoAcquity system (Waters, Milford, MA) connected to an Orbitrap Elite hybrid mass spectrometer (Thermo Electron, Bremen, Germany) equipped with a PicoView nanospray interface (New Objective, Woburn, MA). Peptide mixtures were loaded onto a 75 μm ID, 25 cm length C18 BEH column (Waters, Milford, MA) packed with 1.7 μm particles with a pore width of 130 Å, and mixtures were separated using a segmented gradient in 5% to 40% solvent B (acetonitrile with 0.1% formic acid) for 90 min at a flow rate of 300 nl/min and a column temperature of 35°C. Solvent A was 0.1% formic acid in water. The mass spectrometer was operated in the data-dependent mode. Briefly, surveys of full-scan MS spectra were acquired in the orbitrap (*m/z* 350–1600) with the resolution set to 60K at *m/z* 400 and an automatic gain control (AGC) target of 106. The 15 most intense ions were sequentially isolated for HCD MS/MS fragmentation and detection in the orbitrap with previously selected ions dynamically excluded for 90 s. For MS/MS, we used a resolution of 15000, an isolation window of 2 *m*/*z* and a target value of 50000 ions, with maximum accumulation times of 200 ms. Fragmentation was performed with a normalized collision energy of 35% and an activation time of 0.1 ms. Ions with singly and unrecognized charge state were also excluded. The mass spectrometry proteomics data have been deposited into the ProteomeXchange Consortium [35] via the PRIDE partner respository with the dataset identifier PXD002705.

### Global proteomic data analysis

MS raw data files obtained from the LTQ-Orbitrap Elite were set for relative quantification and identification using Proteome Discoverer version 1.4 (PD) (Thermo Fisher Scientific, San Jose, CA). Database searches for each set were performed by the Mascot search engine (v. 2.5) using the following criteria: decoy UniProt *E*. *coli* database, MS peptide tolerance as 10 ppm, MS/MS tolerance as 0.05 Da, trypsin digestion allowing 2 missed cleavages with variable modifications (methionine oxidation, cysteine carbamidomethylation), and fixed modifications (N-terminal TMT6plex, lysine TMT6plex). Only those peptides with IonScores exceeding the individually calculated 99% confidence limit were considered to be accurately identified. PD TMT-labeled quantitation between *E*. *coli* strains was performed using normalized protein intensities. Each candidate was scored based on relative abundance (RA) with at least two ratio counts to acquire the normalized protein intensity (LFQ intensity) and compared against the control sample. Finally, the proteins identified from the database and common contaminants like keratin and trypsin were eliminated from the list of quantified proteins. To determine the general function of proteins in *E*. *coli*, we referred to the annotated data from UniProt (www.uniprot.com).

### *In vivo* complementary overexpression and deletion assay

Essential genes that were highly downregulated in the OE-NrdR strain were complementarily overexpressed under the NrdR overexpression background to test possible rescue in terms of bacterial fitness. Six individual clones of essential genes (Appa, ThiL, PolA, Eno, FbaA and Pgk) downregulated by NrdR overexpression were obtained from the ASKA library in a plasmid pCA24N (Cam^+^) [36], and were ectopically overexpressed individually in a WT *E*. *coli* BW25113 [27] carrying an NrdR overexpression construct in the pETDuet (Kan^+^) vector. The double transformants were selected at 30°C on agar plates that contained LB, 50 μg/ml of kanamycin and 30 μg/ml of chloramphenicol. Fresh overnight cultures were grown from each individual colony, and then the *A*600 of each culture was adjusted to 0.2 by addition of LB. Serial dilutions (10-fold) of these cultures were spotted onto agar plates that contained LB, 0.5 mM IPTG, 50 μg/ml kanamycin and 30 μg/ml chloramphenicol, and then incubated for 10 hours at 37°C or 42°C to evaluate bacterial fitness. As controls, a WT *E*. *coli* strain with two empty plasmids and the DnaK overexpressing pETDuet vector were also subjected to the fitness assay. The above six individual overexpressing genes in *E*. *coli* were also subjected to bacterial growth fitness assay as described above, but in the absence of NrdR repression to account for the effect caused by NrdR.

Similarly, non-essential genes that were downregulated while NrdR was overexpressed were deleted individually under the NrdR overexpression background to test whether this caused any deterioration in bacterial fitness. Strains of seven single non-essential deleted genes (NarH, TrxA, YgfK, CadA, YtfQ, RuvB and YdbK) were obtained from the Kieo collection (the isogenic mutants from WT *E*. *coli* strain BW25113) [27, 28], and were transformed with the NrdR overexpression construct in pCA24N (Cam^+^) vector. Transformants were selected at 30°C on agar plates that contained LB, 50 μg/ml of kanamycin and 30 μg/ml of chloramphenicol. Fresh overnight cultures were grown from each single colony, and then the *A*600 of each culture was adjusted to 0.2 by addition of LB. Serial dilutions (5-fold) of these cultures were spotted onto agar plates that contained LB, 0.5 mM IPTG, 50 μg/ml kanamycin and 30 μg/ml chloramphenicol, and then incubated for 10 hours at 37°C or 42°C to evaluate bacterial fitness. As controls, a WT *E*. *coli* strain with above two empty plasmids and the DnaK-deletion (ΔDnaK) were also subjected to the fitness assay. As described above, the single deletion *E*. *coli* mutants of these seven genes were also tested individually for bacterial growth fitness in the absence of NrdR repression.

### Transmission Electron Microscopy

The exponential phase *E*. *coli* cultures of WT, ΔNrdR and OE-NrdR were grown at 37°C with agitation and allowed static growth for 2 hours. 5 μl of indicated bacterial cultures in water were placed on glow discharged 300 mesh copper grids (Electron Microscopy Sciences, Hatfield, England) for 1 min, and negatively stained for 30 sec with 2% Uranyl formate. The grids were examined at ×21,000 magnification with a Tecnai G2 Spirit TWIN (FEI^™^) transmission electron microscope.

### Cell culture and bacterial infection

Cell culture and bacterial infection assays were performed as described previously [37]. In brief, monolayers of human intestinal epithelial cells (Caco-2, ATCC^®^ HBT-37^™^) were grown as per the specified ATCC culture conditions to a confluence of 5 × 10^6^ cells/well and were then infected with wild type *E*. *coli* isolated from a gastroenteritis-expressing virulence factor and an autotransporter [37], and with the ΔNrdR and OE-NrdR strains. The ΔNrdR strain was prepared by replacing the kanamycin antibiotic marker by homologous recombination and the IPTG-inducible NrdR over-expressing *E*. *coli* was generated as described earlier. About 7 × 10^8^ bacterial cells in 1 ml were used to infect the 95% confluent monolayer of mammalian cells, and uninfected mammalian cells were used as a control. After three-hour incubation with indicated bacterial cultures at 37°C, the monolayers were washed twice with phosphate-buffer saline (PBS; pH 7.2) to remove unbound bacteria. Adherent bacteria were counted by measuring colony forming units (CFU/ml) by serially diluting the cultures as described in [37]. The observed bacterial adherence and CFUs from triplicate results were plotted.

For direct observation of bacterial adhesion, the above-described individual *E*. *coli* strains were ectopically expressed with GFP (pTric3X plasmid, a kind gift from Prof. Yu-Chan Chao’s lab of the Institute of Molecular Biology, Academia Sinica) and then infected into the monolayer of epithelial cells grown on glass cover slips. Cells were incubated at 37°C for three hours and the monolayers were washed with PBS buffer to remove the unbound bacteria. Infected epithelial cells with adherent bacteria were visualized at ×1000 for GFP fluorescence at 475nm wavelength under an FITC filter and using a Zeiss^™^ Observer Z.1 microscope and were imaged using a CCD camera run through AxioVision software. Image merging was performed with ImageJ software. The observed green fluorescence is directly proportional to the number of bacteria adhering to host mammalian cells.

## Results

### Multiple sequence alignment, purification and DNA binding activity of *Ec*NrdR

Comparative studies have shown that NrdR possesses conserved domains in all bacterial species [9, 10, 23]. Positioning of NrdR recognition sequences in the genome shows some variation among bacterial species, and this is particularly marked between Gram-positive and -negative bacteria [8, 9]. To identify the key variation in target recognition, we aligned the NrdR protein sequence from different bacterial classes to find the most conserved and non-conserved regions using ClustalW2 (S1 Fig). Our sequence alignment of NrdR from a broad range of species led to three important observations: 1) the alignment showed highest homology in the N-terminal domain, which is the primary DNA-binding region, and not in the well-known unique ATP-cone domain; 2) although previous reports have noted the importance of conserved N-terminal Cys residues in Zn^+^ binding [14], we observed that Cys residues were arranged in characteristic CPxC and CxxC motifs in all classes of bacterial species; and 3) we noticed that Arg residues (26–29) were highly conserved, exhibiting a unique arginine-motif (R^4^) that might be important for interactions with DNA substrate, as Arg residues are known to play a key role in DNA-protein interactions [38]. In addition, the two cysteine motifs may help in coordination of Zn^+^ ions to stabilize the protein for effective DNA binding through its conserved Arg residues. We speculate that the observed sequence divergences in the ATP-cone domain might give rise to the variation in target gene recognition by NrdR or its diverse functions in different bacterial species. Previous results indicated the binding of nucleotides to the ATP-cone domain and its consequent weak DNA interactions also support our speculation [13].

**Fig 1:**
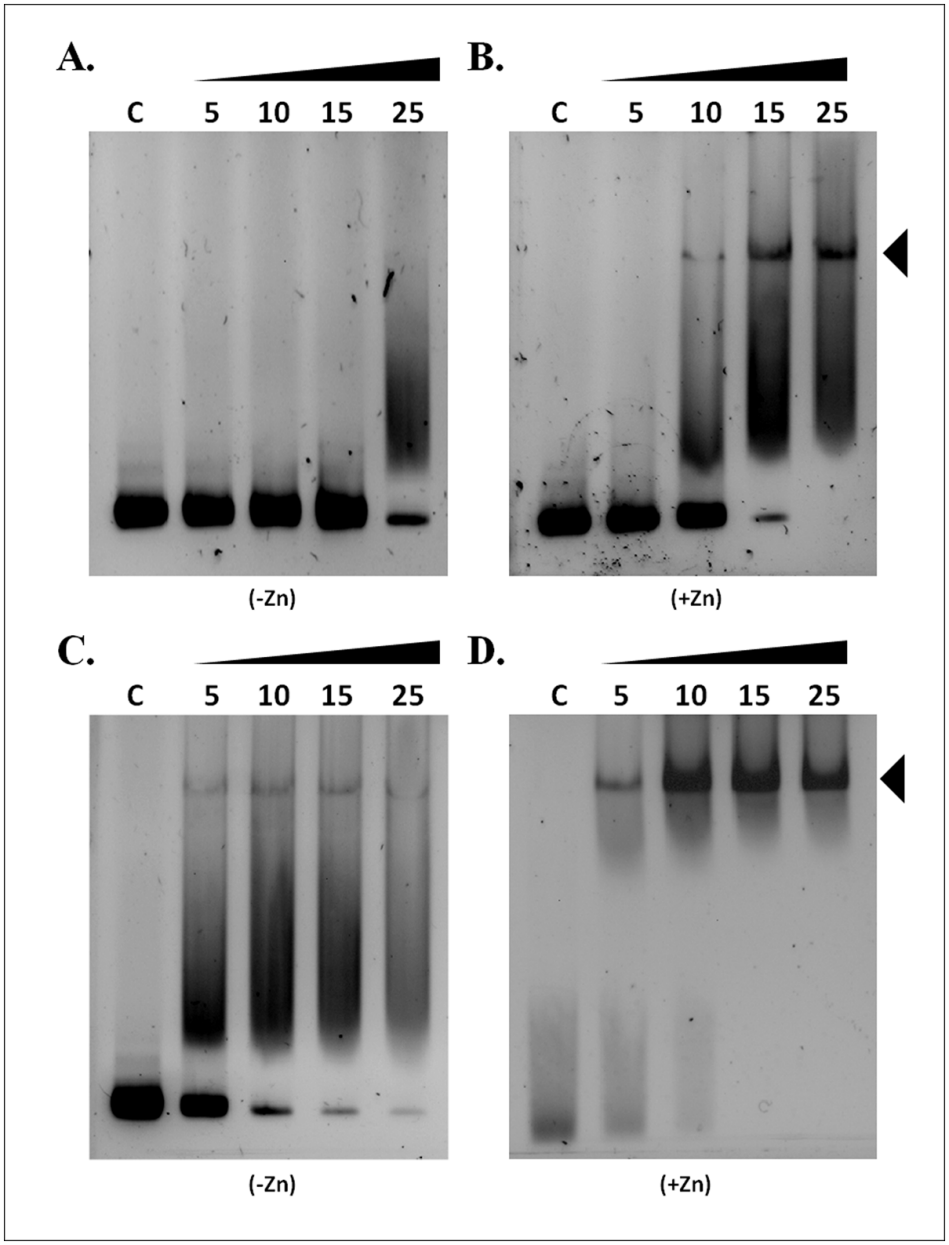
Gel retardation assay of NrdR oligomeric and apo protein against the *nrdAB* promoter. **(A and B)** As prepared NrdR, and **(C and D)** apo-form protein was titrated against *nrd*AB promoter DNA substrate. DNA binding activity of NrdR is shown without (left panel) and with (right panel) supplementation of ZnSO_4_. The NrdR protein concentrations of 5, 10, 15 and 25 μM are shown in corresponding lanes. Filled triangles denote the positions of protein-DNA complex.

The conserved N-terminal zinc-binding region, but not the classical Zn finger, in NrdR is proposed to be important for zinc coordination and for the stability of the NrdR protein [14]. This led us to examine NrdR DNA binding activity in the presence of auxiliary zinc. For this study, we initially purified *E*. *coli* NrdR in oligomeric form (as prepared) (S2A Fig), which is known to bind large amounts of ATP, and dATP, hinders DNA binding [11, 14]. To obtain “apo-form” NrdR, protein unfolding-refolding was performed as described earlier (see Materials & Methods) [11] (S2B Fig). The corresponding elution peaks of the purified “as prepared” (nucleotide bound) and “apo” (refolded) proteins were consistent with previous purification studies, confirming the presence of NrdR protein in these two different conformational conditions. The purified proteins were then tested for DNA binding activity in the presence or absence of auxiliary zinc (Fig 1). The nucleotide bound NrdR showed weak DNA binding activity even at 25μM of NrdR protein in the absence of supplementary zinc. However, addition of auxiliary zinc influenced DNA binding activity, but still predominantly at higher protein concentrations (Fig 1A and 1B). Although refolded or apo-form NrdR showed DNA binding activity without auxiliary zinc, addition of zinc further enhanced DNA binding ability (Fig 1C and 1D). The observed DNA binding activity of NrdR is directly proportional to increasing concentrations of the protein. These results suggest that zinc binding to the N-terminal domain promotes the DNA binding activity of NrdR. Thus, we speculated that zinc ions could help to modulate the conformation of NrdR for effective DNA recognition or binding. Hence, we next tested the DNA binding activity of NrdR with varying concentrations of zinc, whilst keeping a protein concentration of 25 μM as standard. As shown in S3 Fig, addition of zinc at concentrations >1 μM (but not below) influenced the DNA binding activity of NrdR. However, we noticed that 3 μM of zinc was optimal to induce effective DNA binding by the nucleotide-bound NrdR, as a more stable band-shift was achieved compared to that of 1 and 2 μM of zinc. We also noticed that lower concentrations of zinc (0.1 μM to 0.5 μM) did not greatly influence DNA binding activity.

### NrdR overexpression retards bacterial growth

**Fig 2:**
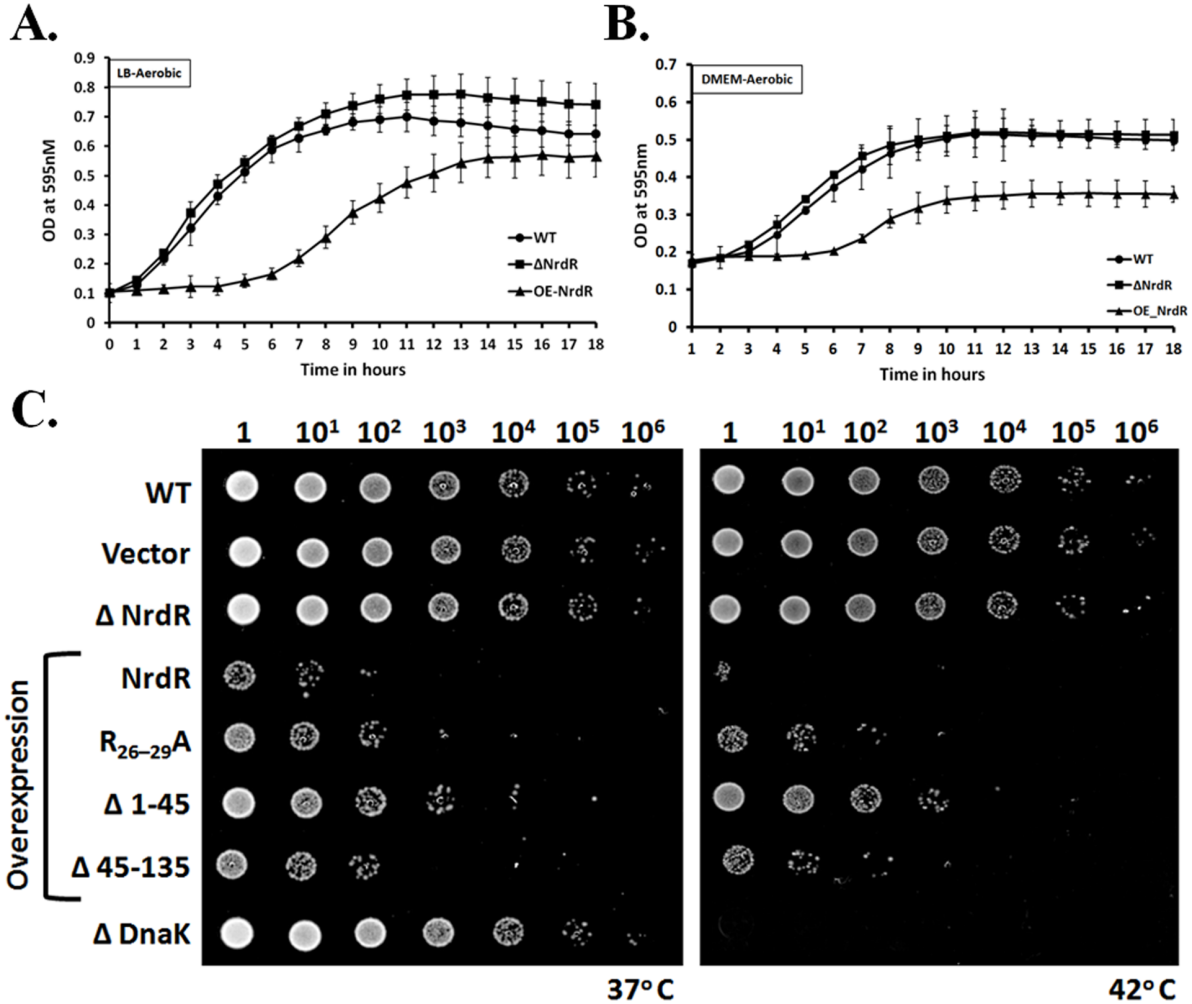
Bacterial proliferation and fitness are inversely correlated with NrdR expression. Synchronous growth curves of WT (closed circle), ΔNrdR (closed square) and OE-NrdR (closed triangle) *E*. *coli* under aerobic conditions in LB medium **(A)** and in cellular medium (DMEM) **(B)**. Growth curves were recorded for 18 hours using the Tecan ^®^ Synchronous Growth Reader with measurements taken at OD 595 nM. **(C)** Growth viability assay of NrdR strains. Serial dilutions of indicated fresh *E*. *coli* cultures were spotted onto agar plates containing LB and 0.5 mM IPTG and incubated at 37°C or 42°C overnight. Bacterial viability was significantly reduced in cells overexpressing NrdR at 37°C, and was lethal at 42°C, but not for ΔNrdR mutants at both temperatures. The Arg-motif mutant and the deletion mutants of the N-terminal and ATP-cone domains show partial rescue in the bacterial fitness defect caused by NrdR overexpression. Empty vector and the ΔDnaK strain were used as negative and positive controls, respectively. Dilution factors for the *E*. *coli* cultures are labeled over the panels.

Previous studies have shown that loss of NrdR does not alter bacterial growth patterns in LB medium [8, 9, 23]. Hence, we next extended our comprehensive study to examine cell proliferation in response to elevated levels or overexpression of NrdR. To evaluate the phenotypes, the ΔNrdR and OE-NrdR strains were tested for growth in LB medium under aerobic conditions (Fig 2A). Interestingly, overexpression of NrdR caused a significant delay in growth, with initiation of its exponential phase only occurring after 5 hours of incubation (Fig 2A). Plasmid isolation and restriction digestion studies from the steady-phase cultures of OE-NrdR showed that plasmid loss or *nrdR* gene rearrangement did not occur (data not shown) and so are not the causes of strain outgrowth after the extended lag phase. Instead, this outgrowth after an extended lag phase might result from eventual bacterial stabilization following NrdR repression, even though it does not attain the high OD of WT at the exponential phase. In contrast, growth profiles of ΔNrdR were normal and consistent with previous observations in different bacterial species [9] in that the growth pattern and time to attain the exponential phase was comparable with the WT *E*. *coli* strain. We next examined *E*. *coli* growth patterns in DMEM (mammalian cell culture medium) supplemented with 10% FBS under aerobic conditions to mimic the host cell environment (Fig 2B). The results were consistent with our studies in LB medium, showing significant growth retardation of the OE-NrdR mutant but not for the NrdR-deletion mutant.

In order to assess the specific effects of NrdR overexpression, we performed the growth assay with a negative control by overexpressing the neutral protein β-galactosidase from LacZ operon in both the WT and the NrdR-deletion background in the presence of 0.5 mM IPTG (S4A Fig). In comparison with the WT and NrdR-deletion strains carrying the empty vector, the overexpression of β-galactosidase caused some degree of early growth retardation for two hours in the lag phase. However, the β-galactosidase overexpressing WT and ΔNrdR strains quickly regained growth and entered the exponential phase after three hours of incubation in the synchronous growth cultures. In contrast and consistent with our previous observation, overexpression of NrdR with the same concentration of inducer drastically delayed bacterial growth, the exponential phase started from five hours of incubation. These findings indicate that elevated levels of NrdR in early growth phases retards bacterial proliferation. Furthermore, spot and streak plate assays on LB Ampicillin plates containing 0.5 mM IPTG and 40 μg/ml X-Gal with overnight incubation, used to assess bacterial fitness, showed that both WT and ΔNrdR *E*. *coli* strains expressing β-galactosidase did not exhibit an overall growth defect compared to that of OE-NrdR (S5 Fig).

To further corroborate the growth defect caused by NrdR overexpression observed in our growth curve profiles, we performed CFU analysis. As shown in S4B Fig, WT and ΔNrdR *E*. *coli* strains after the initial five hours produced increasing numbers of CFUs with increasing incubation time. By the end of the exponential phase, the WT and ΔNrdR cultures had produced ~9.5E^10^ CFUs/ml. However, the number of CFUs for the OE-NrdR strain did not begin increasing until five hours into incubation and, after 16 hours incubation when the culture had reached the end of the exponential phase, only ~5E^10^ CFUs had been generated. Our growth curves and CFU analysis both suggest that NrdR overexpression or elevated levels hinder bacterial growth during both the lag and exponential phases and act as a negative regulator of bacterial growth. From these observations we hypothesize that wild-type bacterial strains would only begin to express the NrdR repressor during the late exponential phase when the cell needs to regulate or suppress several genes that may no longer be needed. In support of this hypothesis, elevated expression of NrdR has been reported for stationery phases of *E*. *coli* cultures [39] under normal growth. These observations are also consistent with our time-course expression studies and this allowed us to access the degree of NrdR expression and overall protein production in bacteria (S4C Fig). NrdR expression was logarithmically increasing with time but the overall protein levels the cell has not much altered until the initial five hours of incubation in the presence of inducer. The increase in the total cellular protein was observed from the seven hours after the incubation, which suggests the outgrowth of the strain that is relatively consistent with the OD as observed in the synchronous growth (Fig 2).

To further test bacterial viability, we examined the ΔNrdR and OE-NrdR strains for growth fitness. Both these bacterial strains and the WT were serially-diluted and subjected to spot assays by growing them in LB medium containing 0.5 mM IPTG at normal (37°C) and elevated temperatures (42°C). As shown in Fig 2E, NrdR-deletion in *E*. *coli* did not negatively impact cell viability even under the heat shock condition. However, overexpression of NrdR in *E*. *coli* resulted in a fitness defect, with growth being considerably retarded even at 37°C and lethal at the elevated temperature (Fig 2E). Lethality under this physiological scenario could be due to the combined effect of the elevated temperature and higher rates of gene suppression.

NrdR possesses a highly-conserved and unique Arg-motif (R^4^), but its functional importance has yet to be investigated. We tested whether mutation of this Arg-motif had a positive feedback on bacterial growth. We individually investigated an *E*. *coli* strain with a mutated NrdR Arg-motif for growth fitness at 37 and 42°C, as well as N-terminal deletion (residues 1–45) and ATP-cone domain deletion (residues 45–135) strains. As shown in Fig 2C, the Arg-motif mutant (R_26-29_A) and the ATP-cone deletion (Δ_45–135_) in NrdR exhibited a trivial improvement in growth/fitness in comparison with the overexpression of native NrdR. This trivial improvement might be due to the fact that arginine residues are involved in DNA-protein interactions [38] and, similarly, ATP-cone domains are known for nucleotide binding and weak DNA interactions [13]. However, the N-terminal deletion mutant (Δ_1–45_) showed a greater improvement in bacterial fitness, even at the higher temperature, which could be due to the defect in DNA binding and gene repression. The partial but not complete recovery in growth for the N-terminal deletion mutant also suggests a possible role for NrdR in other biological functions, either directly or indirectly. The results of our *in vivo* cell viability or fitness assays corroborate our time-course growth curves for individual *E*. *coli* strains, suggesting that elevated levels of NrdR retard bacterial growth and fitness.

### NrdR overexpression causes bacterial aggregation and morphological changes

**Fig 3:**
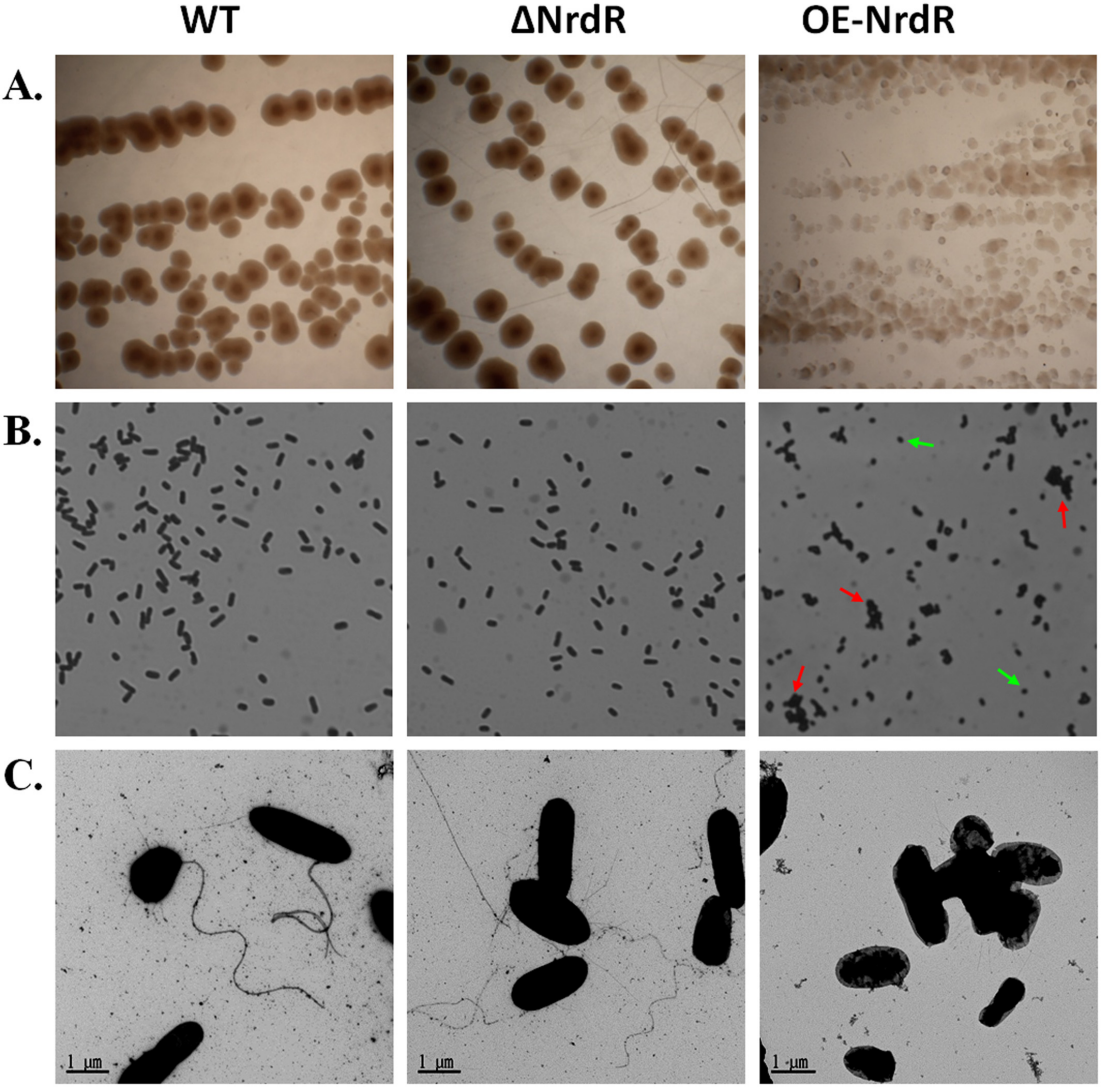
NrdR overexpression influences changes in bacterial morphology. **(A)** Colony morphology of WT, ΔNrdR and OE-NrdR *E*. *coli* strains grown on LB medium. **(B)** Magnification (×100) of *E*. *coli* strains. Overexpression of NrdR resulted in bacterial aggregates (red arrows) and coccobacilli (short rods; green arrows). **(C)** Transmission electron microscopy of the WT, ΔNrdR and OE-NrdR *E*. *coli* strains at high magnification (magnification, ×21,000). Differences in flagella and cell walls among the *E*. *coli* strains can be observed.

As significant growth retardation and heat sensitivity were observed in *E*. *coli* overexpressing NrdR, we next examined the effects of NrdR deletion and overexpression on colony and bacterial cell morphology. As shown in Fig 3A, *E*. *coli* colonies with elevated levels of NrdR were much smaller and more irregular in shape. We also noticed that colony formation took longer. These results agree with our earlier time-course growth assays in liquid cultures (see Fig 2). However, no significant difference was observed in the colony morphologies of ΔNrdR and WT strains. These observations led us to speculate whether the OE-NrdR strain displayed any changes in morphology that might explain the growth retardation and fitness defect. To inspect cell morphology, mid-log phase fresh cultures of the indicated *E*. *coli* strains were initially examined by microscope at ×1000 (Fig 3B). Images of the stained ΔNrdR bacteria did not show any noticeable differences in morphology compared to the WT strain. However, the OE-NrdR *E*. *coli* appeared shorter in length compared to both the WT and ΔNrdR-deletion strains. Furthermore, small bacterial aggregates were seen across several microscopic fields. We exclude the possibility that the observed changes in bacterial morphology or aggregates were due to inclusion body formation, as NrdR is a highly soluble protein and its overexpression does not induce protein inclusions.

To observe the morphological changes more closely, we next examined all three *E*. *coli* strains by transmission electron microscopy (TEM, magnification of ×21,000, Fig 3C). Consistent with our earlier observations at low magnification, TEM images of UF-stained ΔNrdR *E*. *coli* exhibited morphological features relatively similar to WT bacteria. However, in comparison to WT bacteria, ΔNrdR strains did exhibit emaciated flagella. Interestingly, attached flagella were hardly detectable in the OE-NrdR *E*. *coli*, bacterial cells were shorter, and aggregation of cells with deprived cell walls was clearly observed. These observations suggest that NrdR overexpression results in fragile flagella, which might be defect in anchoring them to the bacterial cell wall. These observations indicate that NrdR overexpression can also lead to changes in bacterial morphology and internal protein composition, which may cause bacterial growth retardation and reduced fitness. These phenotypic observations led us to investigate global protein composition following overexpression of NrdR in *E*. *coli*.

### Global protein profiling of *E*. *coli* NrdR-deletion and -overexpression strains

**Fig 4:**
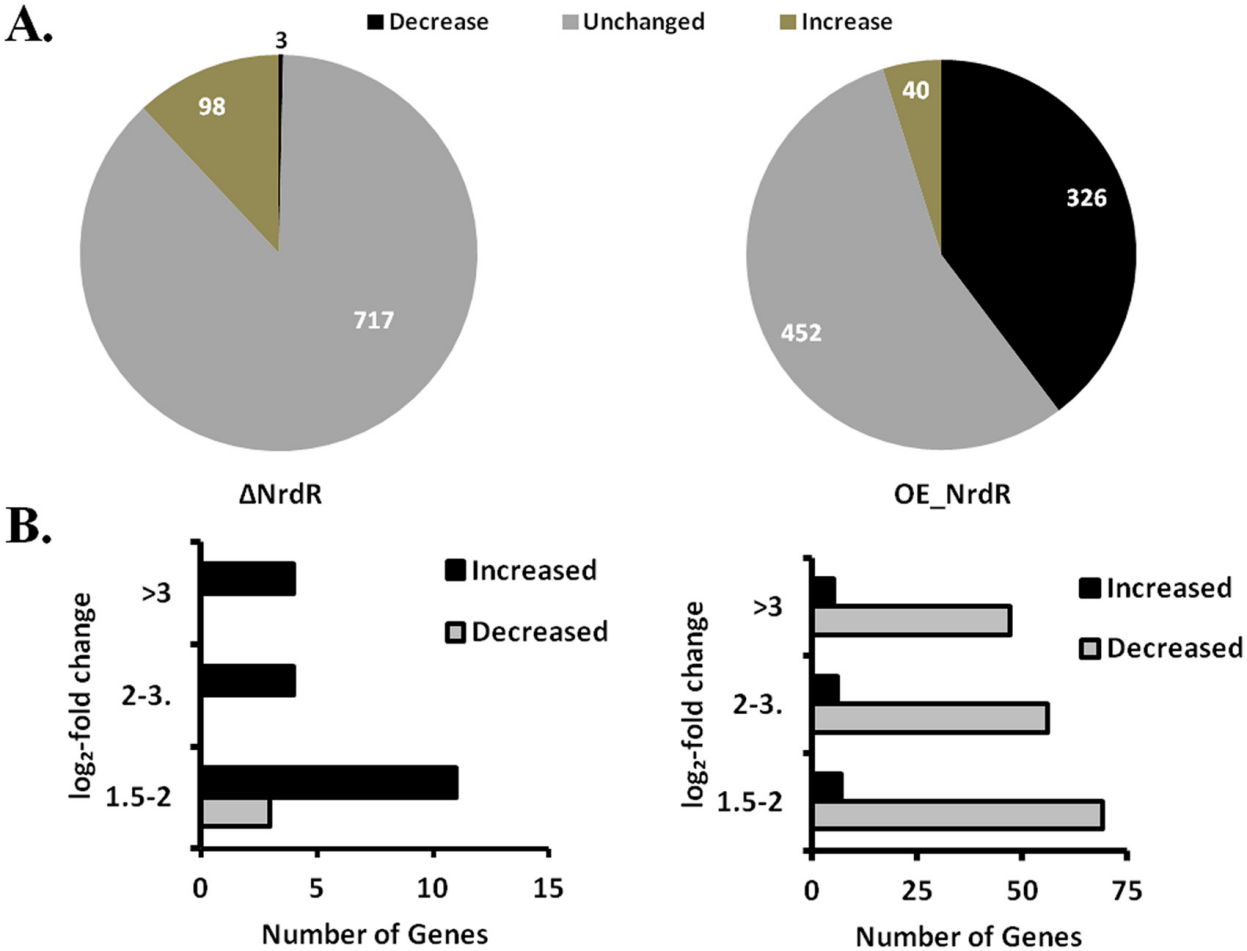
Proteomic response of *E*. *coli* to NrdR-deletion and -overexpression. **(A)** LC-MS/MS resulted in identification of 818 soluble proteins of sufficient abundance to evaluate across strains. TheΔNrdR strain had more upregulated proteins compared to the wild-type, while the OE-NrdR strain had a larger percentage of downregulated proteins. The number of proteins in each category is indicated. **(B)** Selective distribution of upregulated and downregulated proteins according to log_2_-fold change in the ΔNrdR (left panel) and OE-NrdR (right panel) mutants. X-axis represents the number of genes and Y-axis represents ranges of n-fold changes in protein expression.

We had determined that NrdR overexpression retards bacterial growth and causes defective cell morphology, leading us to wonder about the downstream effects of NrdR overexpression. To determine the global proteomic changes caused during NrdR overexpression, we performed comprehensive whole cell protein profiling for the OE-NrdR strain using TMT-labeled LC-MS/MS by LTQ-Orbitrap Elite Mass Spectrometer (Thermo) along with the WT and ΔNrdR strains. Protein extracts from the whole cell lysates of wild-type, ΔNrdR and OE-NrdR strains that are grown as described in our synchronous growth studies were labeled with isobaric TMT tags and subjected to mass spectrometry. Of the more than 4300 known protein-coding genes (as determined by the arithmetic mean of mRNA signals) [33], 837 soluble proteins were detected in the soluble fraction of *E*. *coli* lysates. Among them, 818 proteins were found in abundance for comparative quantification against the WT strain. Our results are comparable with previous mass spectroscopy detections for *E*. *coli* lysates, which cite nearly 900 proteins from soluble fractions [33, 40]. In comparison with the WT strain, the ΔNrdR strain showed differential protein expression for 101 genes (3 downregulated genes and 98 upregulated genes) (Fig 4A), of which only 22 (19 upregulated and all 3 downregulated) exhibited a log_2_-fold change in expression of >1.5-fold (our threshold for significance) (Fig 4B). Interestingly, the OE-NrdR strain showed differential expression for 366 genes; 326 genes were downregulated and 40 were upregulated (Fig 4A). Again, applying our log_2-_fold >1.5-fold threshold, we found that 176 genes were downregulated and only 18 genes were upregulated (Fig 4B) (S6 Fig and S1 Table for a complete list of individual proteins and their expression levels). The higher number of proteins with decreased expression following NrdR overexpression (4.8-fold under a log_2_ scale, as determined from our proteomics analysis) might underlie the observed bacterial growth retardation and lower fitness.

**Fig 5:**
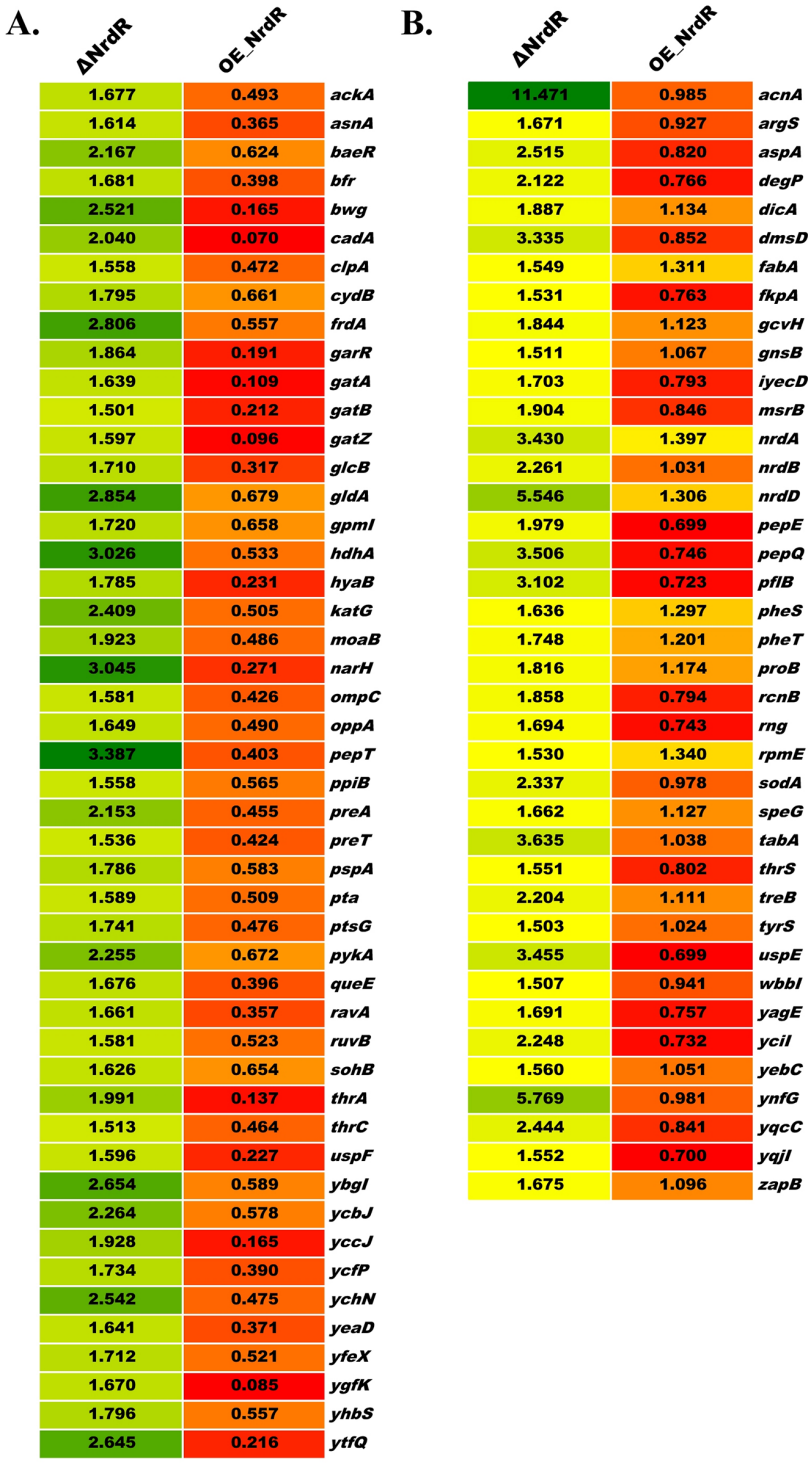
Heat maps representing differential regulation of proteins in *E*. *coli* following NrdR-deletion and -overexpression. **(A)** Protein categories upregulated in the ΔNrdR strain but downregulated in the OE-NrdR strain. **(B)** Protein categories upregulated in the ΔNrdR strain but with no change for the OE-NrdR strain. The color scale indicates differential regulation of protein amounts relative to WT *E*. *coli* control, with upregulation indicated by green shading and downregulation by red. Genes are listed alphabetically.

Based on our global proteome analysis, we next classified the proteins with altered protein expression into two categories: (1) proteins that were upregulated (>1.5-fold) upon NrdR deletion but downregulated (<0.7-fold) while NrdR was overexpressed; and (2) proteins that were upregulated (>1.5-fold) upon NrdR deletion but exhibited no change when NrdR was overexpressed (Fig 5A and 5B). Comparative heat maps showing changes in the expression pattern allowed us to correlate NrdR expression with the corresponding gene fate. This approach revealed a distinct subset of proteins showing inverse relationships with the elevated expression of NrdR in the cell. In addition to decreased expression of several essential genes upon NrdR overexpression, we also found reduced levels of several cell maintenance proteins, including chaperones such as *ravA* and *cadA* (a synergistic ATPase and chaperone known to contribute to bacterial homeostasis in response to stress) [41] (www.EcoCyc.org). Interestingly, expression levels of these chaperones were upregulated in the ΔNrdR strain (Fig 5A). In agreement with a previous study [8], ΔNrdR mutants showed more than a two-fold increase in expression of the RNRs *nrdA*, *nrdD* and *nrdB*. However, we did not observe noticeable upregulation of other RNRs such as class Ib (*nrdHIEF*), which could be due to the compensating role played by the repressor *fur*, a counterpart of *nrdR* [12, 19] in regulating the expression of class Ib. The global proteome changes in response to altered NrdR expression levels suggest that elevated levels of NrdR could also act as a checkpoint for bacterial growth and fitness.

### Complementary overexpression of depleted PolA, ThiL & Eno genes can partially rescue the bacterial fitness defect caused by NrdR overexpression

**Fig 6:**
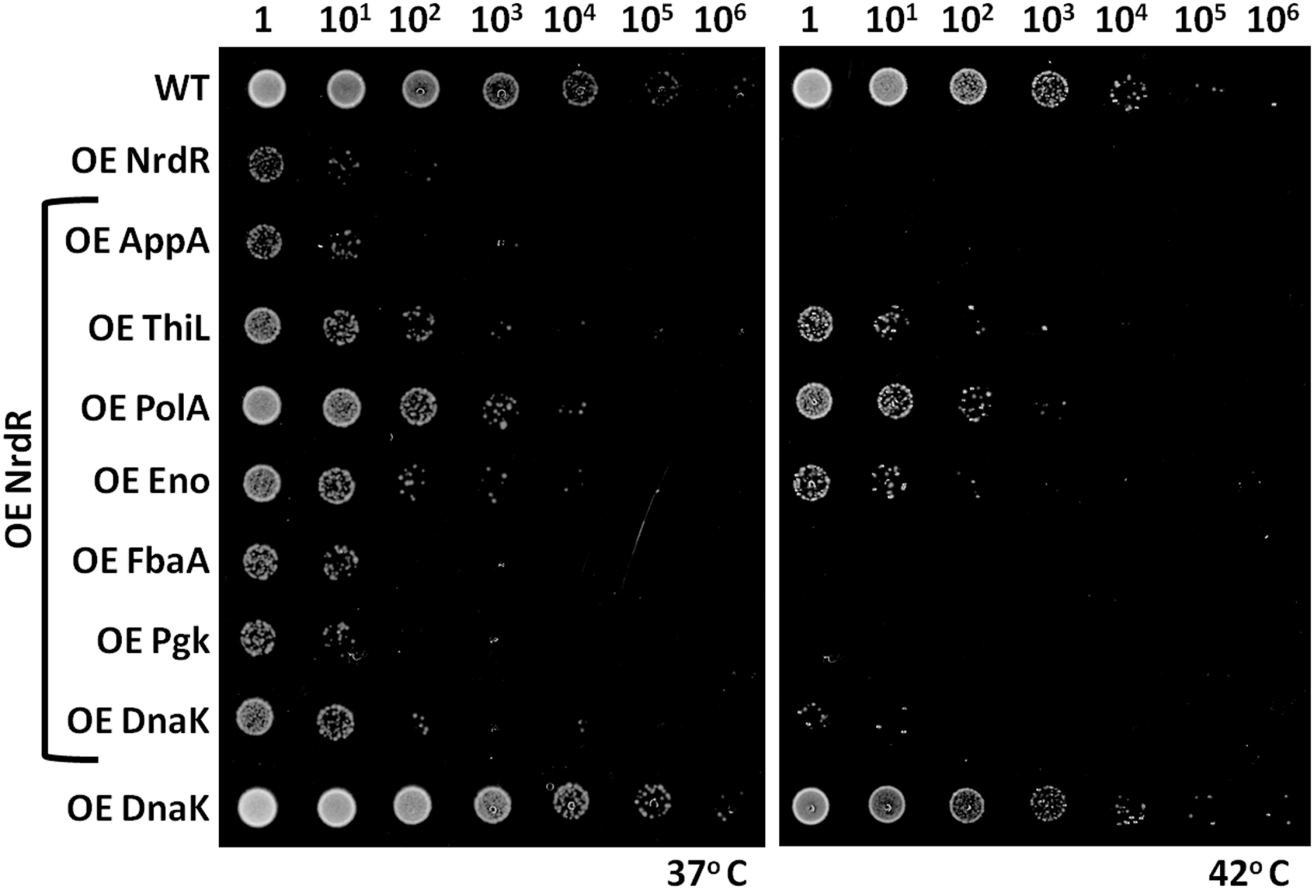
Complementary expression of PolA, ThiL & Eno shows partial rescue of the bacterial fitness defect caused by NrdR overexpression. The downregulated essential genes of Appa, ThiL, PolA, Eno, FbaA and Pgk were complementarily expressed under the NrdR overexpression background to test fitness rescue. Partial rescue of bacterial fitness was observed for PolA, Thil or Eno complementary expression. Complementary expression of genes Appa, FbaA and Pgk resulted in fitness defects resembling those of NrdR overexpression alone and did not result in fitness rescue. Empty vectors pET-duet (Kan^+^) and pCA24N (Cam^+^) were transformed into WT strains as controls. The *E*. *coli* with DnaK overexpression was used as both negative and positive controls by excluding or incorporating NrdR overexpression, respectively. Dilution factors for the *E*. *coli* cultures are labeled over the panels.

Since our global proteome analysis indicated that several essential genes were downregulated in response to nearly five-fold (log_2_ scale) overexpression of NrdR in *E*. *coli* (S1 Table), we next investigated this relationship by means of interaction network analysis and gene complementation. As shown in S7 Fig, our protein interaction network analysis using Cytoscape 3.0.2 and String 9.1 and BioGrid database showed NrdR interaction with essential genes such as RibC, PolA, FtsZ, RibD and ThiL, in addition to RNRs and other non-essential proteins [10, 22, 42]. Interestingly, our proteome analysis also revealed downregulation of PolA and ThiL (-1.5-fold at the log2 scale), as well as for other essential genes not found in our NrdR interaction network. Hence, we tested whether complementary expression of the downregulated essential genes might help to rescue the bacterial fitness defect caused by NrdR overexpression. To do this, we performed individual complementary overexpression of six highly-downregulated essential genes (Appa, ThiL, PolA, Eno, FbaA, Pgk) under the NrdR overexpression background in *E*. *coli* to seek any rescue in bacterial fitness (Fig 6). We also individually confirmed that overexpression of these six essential genes and the control DnaK did not result in an *E*. *coli* fitness or growth defect (S8A Fig) to ensure that the observed change in the global proteome and the bacterial fitness defect is solely caused by the elevated levels of NrdR in *E*. *coli* and are not an artifact of the overexpression. As shown in Fig 6, complementary overexpression of PolA, ThiL or Eno showed partial rescue of the fitness defects caused by NrdR overexpression at both normal and elevated temperatures. Complementary overexpression of ApaA, FbaA or PgK did not rescue the bacterial fitness defect caused by NrdR overexpression. This observation has led us to propose that association of NrdR with PolA, ThiL and Eno might regulate their expression, either directly or indirectly. Both PolA (DNA polymerase I) and ThiL (Thiamine-monophosphate kinase) are key enzymes in the DNA synthesis that facilitates continuity of cell proliferation. Downregulation or suppression of these enzymes might cause fitness defects in bacteria. In support of this hypothesis, optimal expression of PolA, ThiL and Eno is known to be essential for bacterial homeostasis. Furthermore, either reduced levels or mutations in these genes have been implicated in reducing bacterial virulence [43–47]. The partial rescue observed in our complementary overexpression studies is convincing that single gene complementation might be insufficient to overcome the overall effect caused by elevated levels of NrdR in bacterial cells.

### Deletion of downregulated TrxA and YgfK genes exacerbates the bacterial fitness defect caused by NrdR overexpression

**Fig 7:**
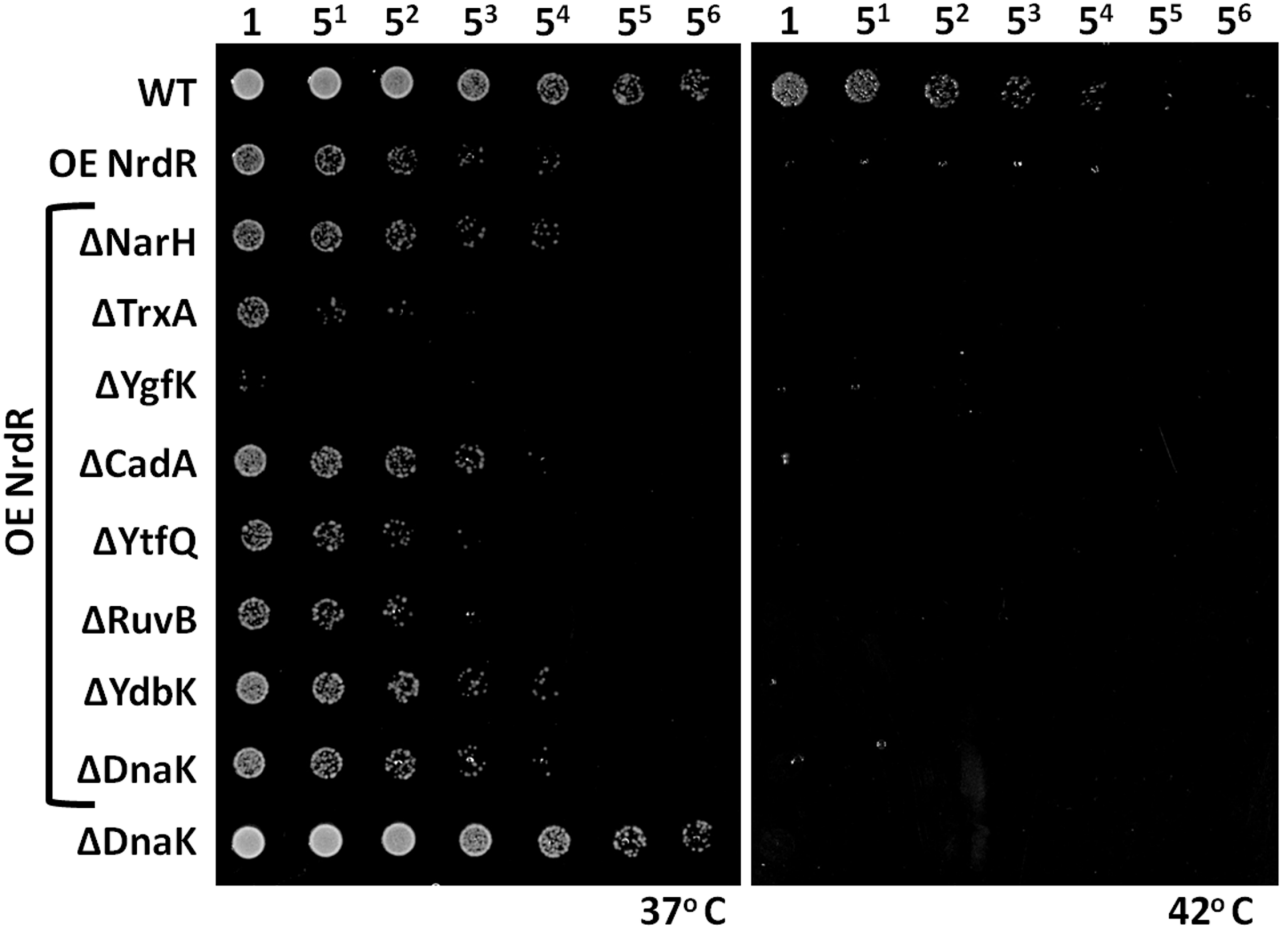
Deletion of downregulated genes upon NrdR overexpression further inhibits bacterial fitness. The highly downregulated non-essential genes NarH, TrxA, YgfK, CadA, YtfQ, RuvB and YdbK were deleted and the corresponding bacterial fitness was studied under the NrdR overexpression background. Deletion of TrxA or YgfK with NrdR overexpression further reduced bacterial fitness compared with NrdR overexpression alone. Deletion of NarH, CadA or YdbK did not result in enhanced reductions in bacterial fitness. Fitness deterioration was not enhanced even with the deletion of YtfQ and RuvB, key genes in DNA damage response. Empty vectors of pET-duet (Kan^+^) and pCA24N (Cam^+^) were transformed into WT strains as control. The ΔDnaK *E*. *coli* strain was used both as negative and positive controls by excluding or incorporating NrdR overexpression, respectively. Dilution factors for the *E*. *coli* cultures are labeled over the panels.

Our proteomic studies showed that increased levels of NrdR also caused downregulation of several non-essential bacterial genes. We tested whether deletion of downregulated non-essential genes upon NrdR overexpression could further inhibit the bacterial growth or exacerbate the fitness defect. We did not use the same study design for non-essential genes as for essential genes, as deletion mutants of these latter are not viable. Previous studies have reported NrdR’s physical or genetic association with the non-essential proteins GlyS, NusB, TrxA, YdbK, RibD and YfhM, and some of them were also identified from our protein interaction network analysis using Cytoscape 3.0.2 and String 9.1 software [19–22, 42, 48] (www.string-db.org). However, concerning non-essential genes in our global proteome analysis, we only found decreased levels of YdbK and TrxA, both of which are known to physically associate with NrdR [19, 48]. Hence, we next tested seven highly-downregulated non-essential genes from our proteomics analysis, including YdbK and TrxA, for genetic compatibility or fitness defects by deletion studies under the NrdR-overexpression background (Fig 7). We also confirmed individually that deletion of these seven non-essential genes did not result in defective bacterial fitness or growth (S8B Fig). As shown in Fig 7, in comparison to NrdR overexpression alone, deletion of TrxA or YgfK caused a fitness defect under the NrdR overexpression background. As elevated temperatures are lethal for NrdR-overexpressing bacteria, we could not assess colony formation. Fitness defects of ΔNarH, ΔCadA, ΔYtfQ, ΔRuvB or ΔYdbK individually under the NrdR overexpression background were much less pronounced compared to those of ΔTrxA and ΔYgfK, and were similar to OE-NrdR alone. Even though YdbK interaction with NrdR has been demonstrated in pulldown experiments [48], our study did not reveal a role for it in regulating the effect caused by NrdR overexpression. Conversely, in support of known evidence for a TrxA interaction with NrdR [19], we clearly observed an exacerbating effect on fitness of the TrxA deletion under the NrdR overexpression background. Therefore, although enzymes like TrxA, RuvB and YgfK are non-essential genes, we found their presence even at reduced levels was necessary under the NrdR overexpression background, since deletion under this phenotypic background caused adverse effects in terms of bacterial fitness. We anticipate that, in addition to the association of NrdR with TrxA, further functional cascades are either directly or indirectly regulated through expression levels of NrdR protein.

### Elevated NrdR levels in *E*. *coli* decrease adhesion to intestinal epithelial cells

**Fig 8:**
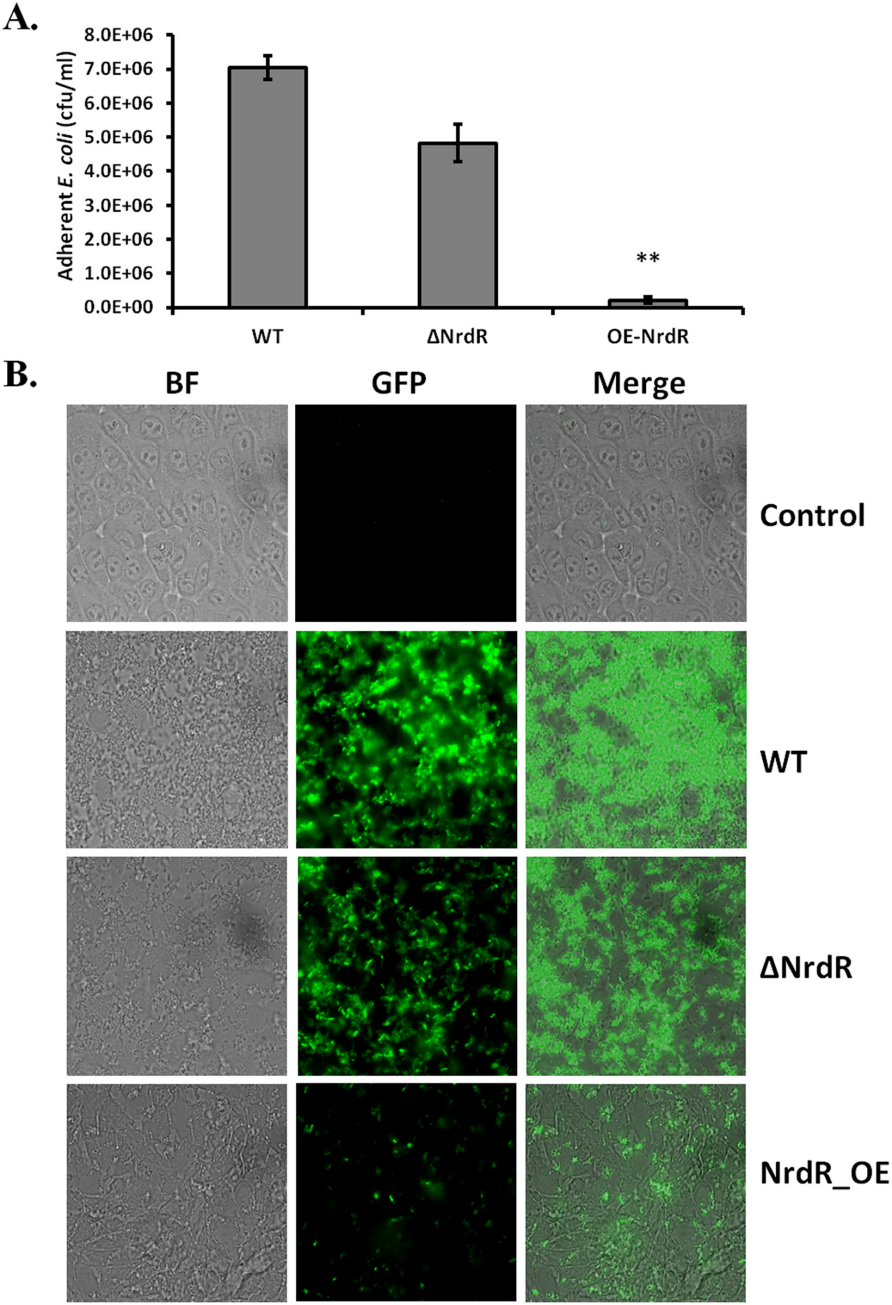
Bacterial NrdR regulates adhesion to mammalian epithelial cells. **(A)** Human epithelial cells in culture were infected with WT, ΔNrdR and OE-NrdR *E*. *coli* strains. Adherent bacteria were counted 3 hours after infection. Results are reported as CFUs/ml. **(B)** Direct observation of GFP-tagged WT, ΔNrdR and OE-NrdR *E*. *coli* adhering to mammalian cells by fluorescent microscopy at ×1000 magnification. The GFP signals were observed under the FITC filter of a UV laser at 475nm.

To evaluate the effect of NrdR overexpression on host-pathogen competitiveness or virulence, we next examined adherence of gastroenteritis-isolated wild-type *E*. *coli* and the NrdR-deletion and -overexpression strains prepared of the same genetic background to human intestinal epithelial cells. Measurements of colony-forming units (CFU/ml) of these strains are shown in Fig 8A. Though both the ΔNrdR and OE-NrdR *E*. *coli* strains showed decreased host cell adhesion in comparison to WT, a more than eight-fold reduction in host cell adhesion was found for the OE-NrdR strain compared to WT, as opposed to the less than 2-fold reduction between ΔNrdR and WT strain. The measured CFUs/ml for the OE-NrdR and ΔNrdR strains were 8 x 10^5^ and 5 x 10^6^, respectively. This host cell binding assay suggests that overexpression of NrdR causes a significant decrease in bacterial virulence in addition to a fitness defect.

To further corroborate this effect and to reveal the physiological basis for the loss of adhesion with respect to altered NrdR expression levels, we introduced a GFP plasmid to the WT, ΔNrdR and OE-NrdR strains to directly observe bacterial adhesion following infection of cultured epithelial cells. Using confocal microscopy, we observed that the WT bacterial cells were able to efficiently adhere to the host cells (Fig 8B). Although fluorescence intensities corresponding to cell adhesion were reduced in the ΔNrdR strain, loss of adhesion was much greater for the OE-NrdR strain, with very low GFP signal corresponding to extremely low levels of cell adhesion. Relative fluorescence intensities for WT, ΔNrdR and OE-NrdR *E*. *coli* strains were 148 ± 43, 97 ± 24 and 15 ± 6 units (log_10_ scale), respectively (S9 Fig). These direct observations agree with the diminished CFU counts of the ΔNrdR and OE-NrdR strains. As a proxy for pathogen-host interactions, the results of our cell adhesion assays strongly suggest that NrdR overexpression in the early growth phase might also diminish bacterial virulence and cause defects in host cell adhesion. Coupled with our proteomic data, these observations lead us to propose that elevated expression of NrdR could be a suitable means to retard bacterial growth and fitness and to induce morphological changes leading to impaired host cell adhesion and bacterial virulence.

## Discussion

The transcription repressor NrdR has been implicated in the regulatory expression of various RNRs and, recently, in bacterial chemotaxis through an unknown mechanism [8, 10]. This study extends our understanding of how elevated expression or active abundance of NrdR exerts control over bacterial growth, fitness and virulence. The observed overall decrease in the global proteome of *E*. *coli* in response to increased levels of NrdR reveals diminished bacterial fitness and homeostasis (see Fig 2E). Hence, we speculate that NrdR expression in the bacterial cell could be tightly controlled and might be initiated only during the late exponential or stationery phases of bacterial growth, or under conditions that require growth arrest (such as starvation), in order to maintain cell homeostasis by decreasing mRNA synthesis. This is supported by previous observations that RNR levels remain constant and then gradually depleted after the late exponential phase [12]. The scenario of increased NrdR abundance has also been reported from experiments on bacterial growth at the stationery phase and during mRNA abundance [26, 39]. It is also speculated that there is a feedback mechanism between the cellular nucleotide pool (rNTPs and dNTPs) and NrdR expression to control bacterial growth and DNA replication. Optimum levels of dNTPs are essential for DNA synthesis, which leads to bacterial growth. As NrdR is a repressor of RNRs, which convert rNTPs to dNTPs, we cannot exclude the possibility that the extended lag phase in the OE-NrdR strain is caused by reduced levels of dNTPs. However, our complementary overexpression and deletion studies showing NrdR’s regulation on other genes suggest the possibility that our observed phenotypes might also be derived from other mechanisms. Early expression of NrdR might lead to defective cells, as a number of genes or their respective cascade proteins might repressed or affected either directly or indirect manner. In support of this hypothesis, NrdR is known to associate with several other bacterial proteins such as TrxA, ThiL, GlyS, RibD, PolA, YdbK and NusB, in addition to the RNRs [19, 20, 22, 42, 48]. Our experimental results from both complementary overexpression and deletion studies under the NrdR overexpression background also supports the possible regulation of some of these proteins by NrdR expression, which may influence bacterial fitness and virulence.

Interestingly, as observed in our global proteome analysis (Fig 4) and the respective physiological studies (Fig 6), increased NrdR expression levels are inversely correlated with *polA* expression [24, 49–52]. In addition, NrdR upregulation has also been observed under the physiological conditions of mRNA abundance, oxidative stress, tryptophan supplementation, DNA enrichment, and in *rpoS* and *trxA* deletion mutants (www.GenExpDB.org/nrdr) [24, 39, 49, 50, 53, 54]. Furthermore, growth retardation has been observed under these conditions, which could be a consequence of increased NrdR levels. Increased NrdR abundance has also been observed in previous transcriptomic studies of altered carbohydrate metabolism and amino acid starvation in *E*. *coli* [52, 55, 56]. This outcome of enhanced transcriptional repression could contribute to retarding bacterial growth by inhibiting the synthesis of cell components necessary for maintaining bacterial fitness. These observations suggest that the overexpression of NrdR in our experiments during the early stage of bacterial growth might have exacerbated gene repression and altered protein interactions, thereby exacerbating bacterial fitness defects and growth inhibition. Our physiological experiments also show drastic reductions in host cell attachment of bacterial cells following NrdR overexpression (Fig 8). Previous transcriptomic studies in *E*. *coli* showed decreased levels of NrdR in interactions with macrophages, during bacterial-virus infection, when in minimal medium and when undergoing temporary heat shock [25, 57–59]. The underlying mechanisms by which NrdR expression is monitored and the transcription factors responsible for regulating NrdR are unclear and require further study. Based on previous protein interaction studies and our physiological experiments, we propose that NrdR might have multiple roles in bacteria, which are modulated by a dNTP co-repressor [60] and the nucleotide pool. It is also evidenced that NrdR associate with several other proteins in addition to its regulation of RNRs, and its newly-reported roles in regulating bacterial chemotaxis, motility, topoisomerase levels and its association with thioredoxin [1, 8, 9, 19] extend its diverse functions. This regulation might help in maintaining bacterial homeostasis through altered expression levels, either directly or indirectly.

In summary, our comprehensive study of NrdR-overexpression and -deletion mutants reveal NrdR’s role in regulating bacterial fitness, growth and virulence. Our findings are supported by *in vitro* and *in vivo* physiological experiments, and global proteomic analysis. Our research emphasizes the importance of regulated levels of NrdR for stable bacterial growth and proliferation, particularly at initial stages of growth. The bacteriological applications of triggering NrdR expression to induce fitness defects and virulence during bacterial infection should be studied further.

## Supporting Information

S1 Fig Protein sequence alignment of *Ec*NrdR with 15 different classes of bacterial pathogens. A ClustalW sequence alignment showing identical residues highlighted in green, conserved residues in yellow, least conserved residues in cyan and non-conserved residues left uncolored. The predicted secondary structure of *Ec*NrdR from the Phyre2 server is denoted above the sequence. The unique arginine-rich motif (R^4^ patch) is marked with a red dashed line and the predicted oligomerization cysteine motifs (CPxC and CxxC) are marked in blue dashed lines. The indicated amino acid sequence numbers correspond to *Ec*NrdR. The aligned organisms are Ec: *Escherichia coli*, Sa: *Staphylococcus aureus*, Sat: *Salmonella typhi*, Rbr: *Rhodospirillum rubrum*, Psa: *Pseudomonas aeruginosa*, Nem: *Neisseria meningitides*, Mep: *Methylobacter pelagicus*, Lim: *Listeria monocytogenes*, Lal: *Lactobacillus lactis*, Klp: *Klebsiella pneumonia*, Hai: *Haemophilus influenza*, Yep: *Yersinia pestis*, Clb: *Clostridium botulinum*, Bsu: *Bacillus subtilis*, Stp: *Streptococcus pneumonia*, My: *Mycobacterium sp*. The residues 3–34 belong to Zinc and DNA binding region and the residues 49–139 belong to ATP-cone domain.
(TIF)

S2 Fig SDS-PAGE analysis of Superdex S200 (Gel filtration)-purified *Ec*NrdR. FPLC Superdex 200 gel filtration chromatography of **(A)** un-refolded and **(B)** refolded *Ec*NrdR is shown in blue and green lines, respectively. The reference molecular weight markers are indicated by the red dashed line with corresponding molecular weights. X and Y-axes represent the S200 elution volume and UV absorbance at 260 nm. The inner panel represents the SDS-PAGE of the corresponding S200 fraction of *Ec*NrdR, and the Ni-column-eluted fraction is shown as input before loading onto gel filtration chromatography. The minor band corresponding to ~40 kDa of the molecular weight marker is a dimer form of NrdR as confirmed by peptide mass fingerprinting. More stable dimers were found in the non-refolded protein fraction compared to the refolded protein fractions. About 5–10% of NrdR species were stable dimers even after protein samples were boiled for 10 min in the presence of 5 mM DTT.
(TIF)

S3 Fig Gel retardation assay for NrdR with varying concentrations of zinc. 25 μM of “as prepared” NrdR protein was titrated against *nrd*AB promoter DNA substrate with increasing concentrations (0.5 ~ 5 μM) of ZnSO_4_. Control DNA substrate alone and with protein alone are shown in lanes 1 and 2, respectively.
(TIF)

S4 Fig Overexpression of NrdR retards bacterial growth and lesser CFUs. **(A)** Synchronous growth curves of WT (closed circle), NrdR-deletion (closed square), β-gal-overexpressing WT (open circle), β-gal-overexpressing+NrdR-deletion (open square) and NrdR-overexpressing (closed triangle) *E*. *coli* under aerobic conditions in LB medium. Growth was measured at OD_595_. Growth curves were recorded for 18 hours using the Tecan^®^ Synchronous Growth Reader with measurements taken at OD 595 nM. Data shown represent the mean ± the standard error of three independent experiments. **(B)** Bacterial growth and survival was determined by measuring CFU ml^−1^ at the indicated time-points. (C) SDS-PAGE results showing the time-course over expression of NrdR with 0.5 mM IPTG. The time (in hours) is indicated over each lane.
(TIF)

S5 Fig Bacterial fitness of NrdR over-expressing *E*. *coli* compared with the overexpression of β-galactosidase in WT and ΔNrdR *E*. *coli*. **(A) Spot assay (B) streak plate** showing overexpression of NrdR, but not the neutral protein β-galactosidase from *lacZ*, causes growth reduction. Both assays were performed on LB plates containing 100 μg/ml of ampicillin, 0.5 μM IPTG and 40 μg/ml X-Gal. Overexpression of β-galactosidase in WT and ΔNrdR background strains did not drastically influence growth upon overnight incubation. However, overexpression of NrdR alone retards bacterial growth and proliferation.
(TIF)

S6 Fig Global protein expression patterns in NrdR-deletion and NrdR-overexpression *E*. *coli* mutants. Scatter plot showing the log_2_-fold change in the protein expression of individual proteins for **(A)** NrdR-deletion and **(B)** NrdR-overexpression mutants. Individual spots represent the individual proteins denoted by Uniprot accession numbers. Each blue spot represents an individual gene. The Y-axis shows the log_2_-fold change and the X-axis indicates the proteins as listed in alphabetical order (indicated numbers correspond to the genes as listed in S1 Table). The substantial number of downregulated genes with decreased protein expression in the NrdR-overexpression mutants is noteworthy.
(TIF)

S7 Fig Protein interaction network analysis. (A) Cytoscape 3.0 and (B) String 9.1, showing the experimental evidence view of the protein interaction network for NrdR.
(TIF)

S8 Fig Bacterial fitness caused by the NrdR overexpression is compared with the overexpression of essential genes and deletion of non-essential genes. (A) Essential genes downregulated by NrdR overexpression (Appa, ThiL, PolA, Eno, FbaA, Pgk) were individually overexpressed in the absence of NrdR repression. Individually overexpressed *E*. *coli* strains show comparative fitness with that of WT or control DnaK-overexpressing strains carrying empty pCA24N (Cam^+^) vector at 37°C. The bacterial fitness defect was observed only with overexpression of NrdR alone. (B) Non-essential genes downregulated by NrdR overexpression (NarH, TrxA, YgfK, CadA, YtfQ, RuvB and YdbK) were individually deleted and assessed for bacterial fitness in the absence of NrdR repression. The individual deletion mutants show comparative fitness with that of WT *E*. *coli* or a control strain lacking DnaK at 37°C.
(TIF)

S9 Fig Relative GFP fluorescence from the individual *E*. *coli* attached to host cells. The average fluorescence of host cell adhered WT, ΔNrdR and OE_NrdR *E*. *coli* form the four independent repeats samples were quantified from the ImageJ software and student *t*-test was performed to shows statistical significance *p* = 7.67 E^-07^.
(TIF)

S1 Table Global proteome analysis of NrdR-deletion and NrdR-overexpression strains compared with the wild-type *E*. *coli* strain. The summarized results are tabulated in the order of serial number, UniProt accession number, protein name, description of the protein, n-fold change in expression and log_2_-fold change in expression (ΔNrdR and NrdR-overexpression) compared to WT *E*. *coli*. Genes are listed alphabetically.
(XLSX)
