# Supplementary figures and images for: NrdR Transcription Regulation: Global Proteome Analysis and Its Role in Escherichia coli Viability and Virulence
Source: PLoS One. 2016 Jun 8;11(6):e0157165. doi: 10.1371/journal.pone.0157165 (PMC4898720; doi:10.1371/journal.pone.0157165)

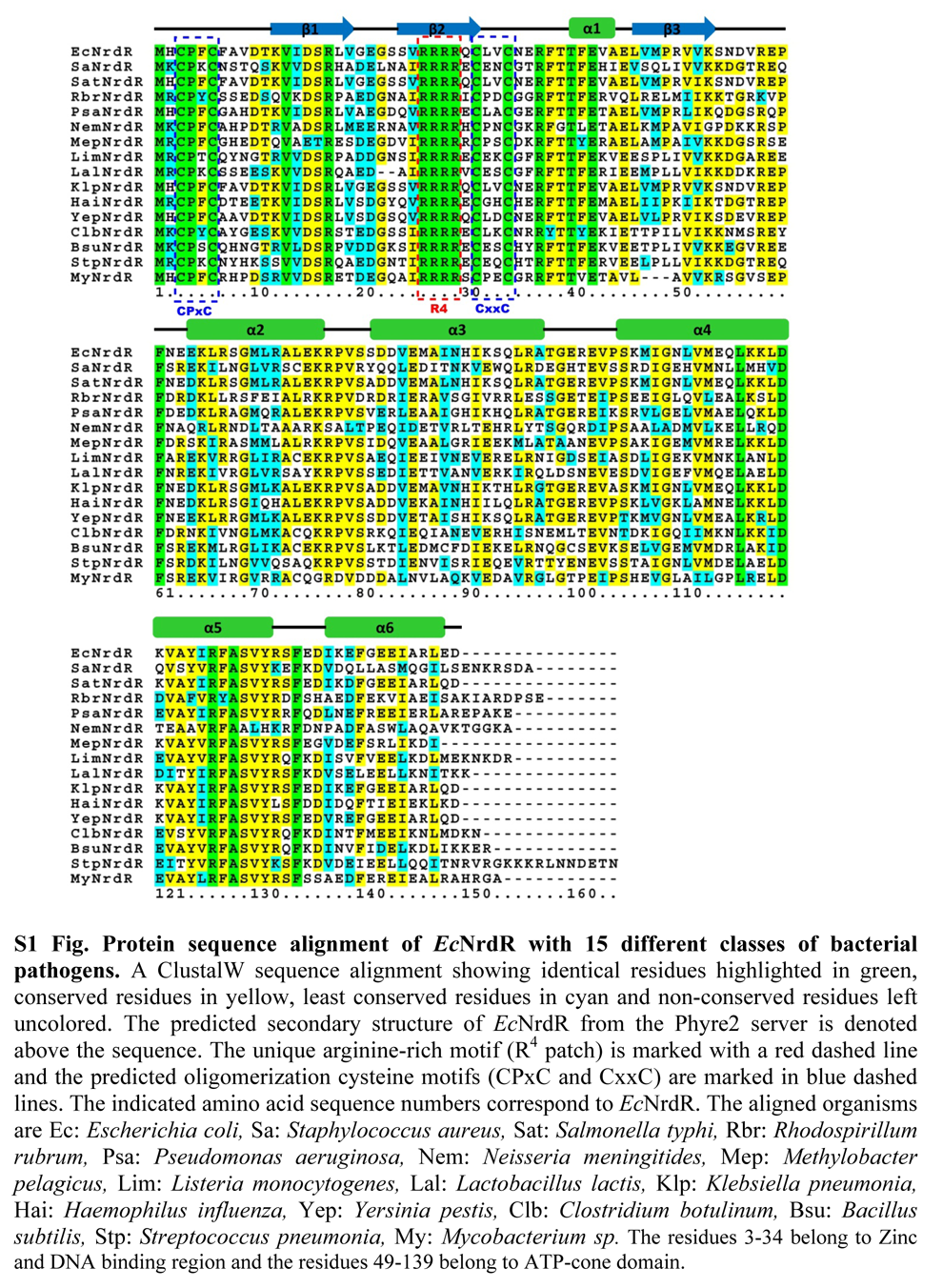

Supplement: S1 Fig — A ClustalW sequence alignment showing identical residues highlighted in green, conserved residues in yellow, least conserved residues in cyan and non-conserved residues left uncolored. The predicted secondary structure of EcNrdR from the Phyre2 server is denoted above the sequence. The unique arginine-rich motif (R4 patch) is marked with a red dashed line and the predicted oligomerization cysteine motifs (CPxC and CxxC) are marked in blue dashed lines. The indicated amino acid sequence numbers correspond to EcNrdR. The aligned organisms are Ec: Escherichia coli, Sa: Staphylococcus aureus, Sat: Salmonella typhi, Rbr: Rhodospirillum rubrum, Psa: Pseudomonas aeruginosa, Nem: Neisseria meningitides, Mep: Methylobacter pelagicus, Lim: Listeria monocytogenes, Lal: Lactobacillus lactis, Klp: Klebsiella pneumonia, Hai: Haemophilus influenza, Yep: Yersinia pestis, Clb: Clostridium botulinum, Bsu: Bacillus subtilis, Stp: Streptococcus pneumonia, My: Mycobacterium sp. The residues 3–34 belong to Zinc and DNA binding region and the residues 49–139 belong to ATP-cone domain. (TIF) [file pone.0157165.s001.tif]

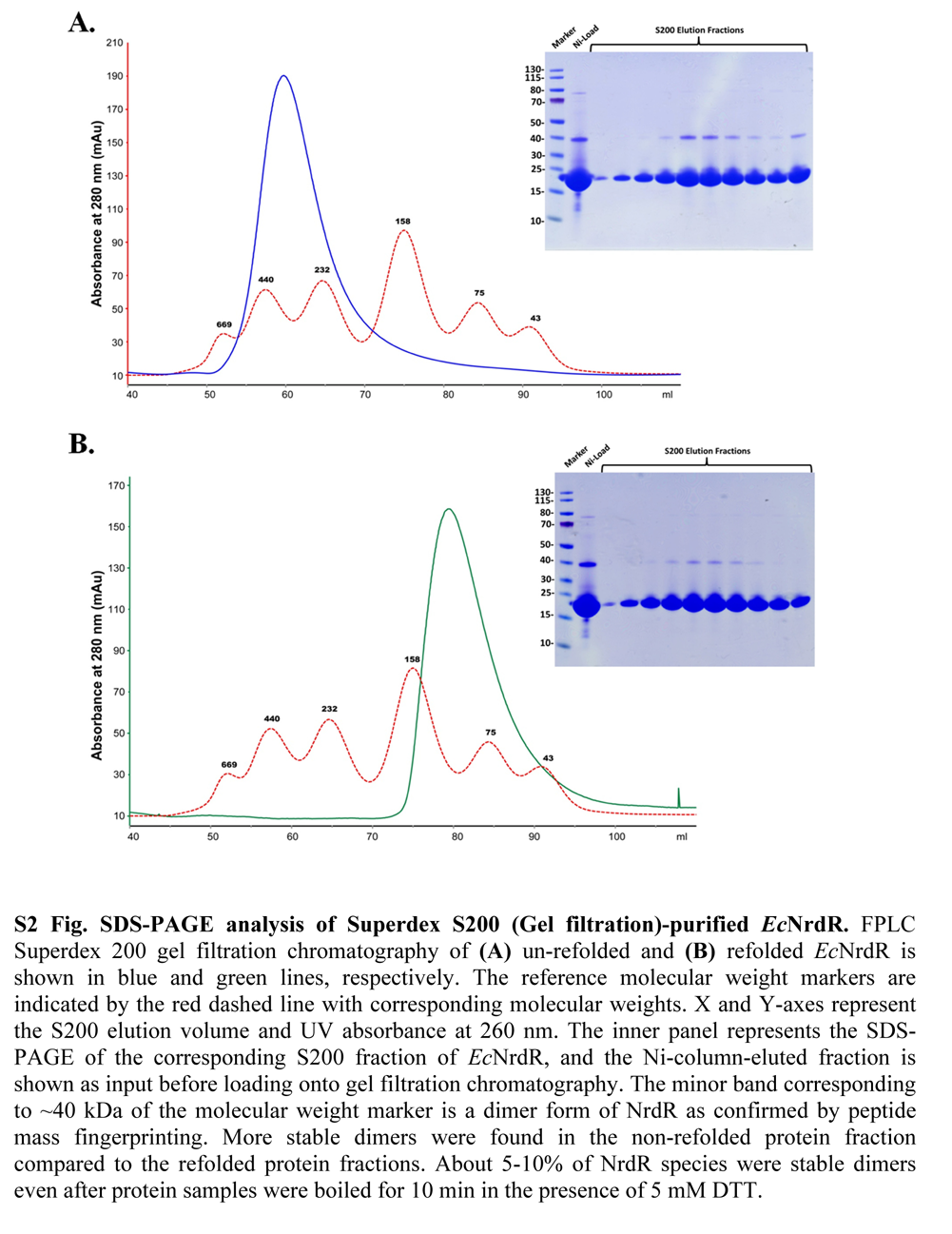

Supplement: S2 Fig — FPLC Superdex 200 gel filtration chromatography of (A) un-refolded and (B) refolded EcNrdR is shown in blue and green lines, respectively. The reference molecular weight markers are indicated by the red dashed line with corresponding molecular weights. X and Y-axes represent the S200 elution volume and UV absorbance at 260 nm. The inner panel represents the SDS-PAGE of the corresponding S200 fraction of EcNrdR, and the Ni-column-eluted fraction is shown as input before loading onto gel filtration chromatography. The minor band corresponding to ~40 kDa of the molecular weight marker is a dimer form of NrdR as confirmed by peptide mass fingerprinting. More stable dimers were found in the non-refolded protein fraction compared to the refolded protein fractions. About 5–10% of NrdR species were stable dimers even after protein samples were boiled for 10 min in the presence of 5 mM DTT. (TIF) [file pone.0157165.s002.tif]

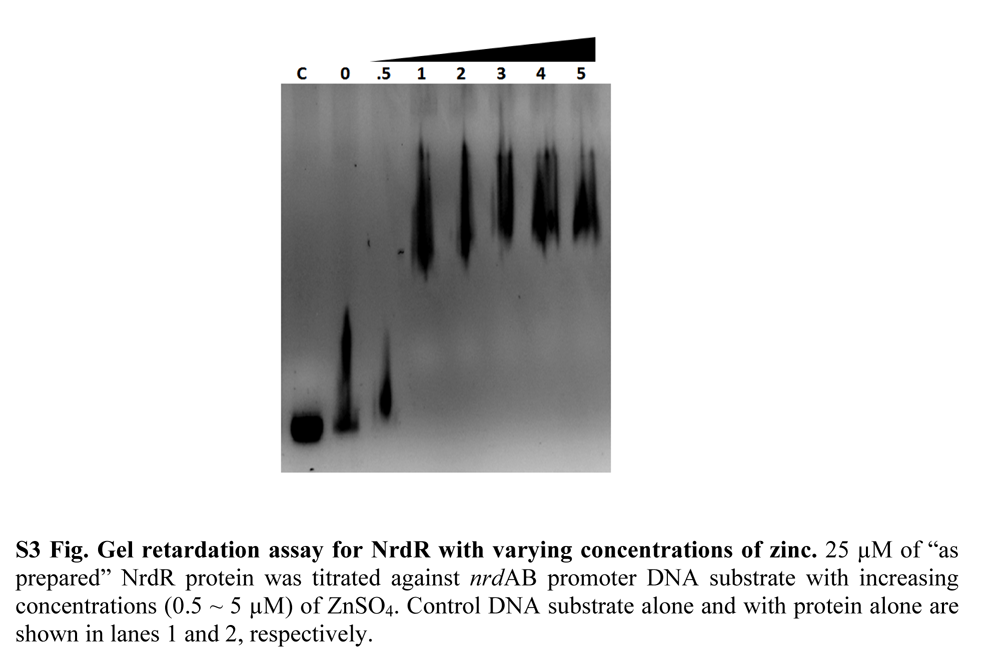

Supplement: S3 Fig — 25 μM of “as prepared” NrdR protein was titrated against nrdAB promoter DNA substrate with increasing concentrations (0.5 ~ 5 μM) of ZnSO4. Control DNA substrate alone and with protein alone are shown in lanes 1 and 2, respectively. (TIF) [file pone.0157165.s003.tif]

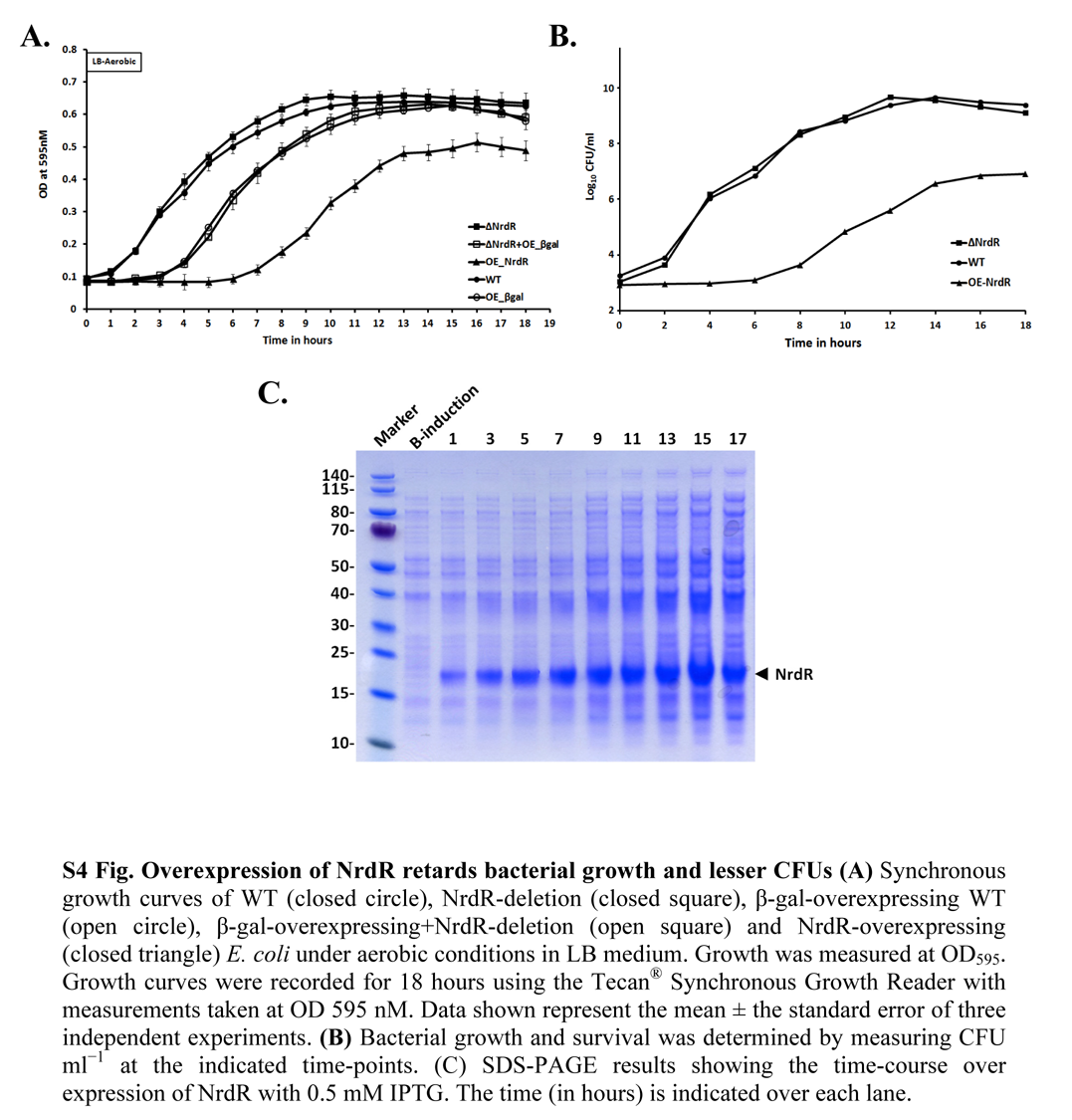

Supplement: S4 Fig — (A) Synchronous growth curves of WT (closed circle), NrdR-deletion (closed square), β-gal-overexpressing WT (open circle), β-gal-overexpressing+NrdR-deletion (open square) and NrdR-overexpressing (closed triangle) E. coli under aerobic conditions in LB medium. Growth was measured at OD595. Growth curves were recorded for 18 hours using the Tecan® Synchronous Growth Reader with measurements taken at OD 595 nM. Data shown represent the mean ± the standard error of three independent experiments. (B) Bacterial growth and survival was determined by measuring CFU ml−1 at the indicated time-points. (C) SDS-PAGE results showing the time-course over expression of NrdR with 0.5 mM IPTG. The time (in hours) is indicated over each lane. (TIF) [file pone.0157165.s004.tif]

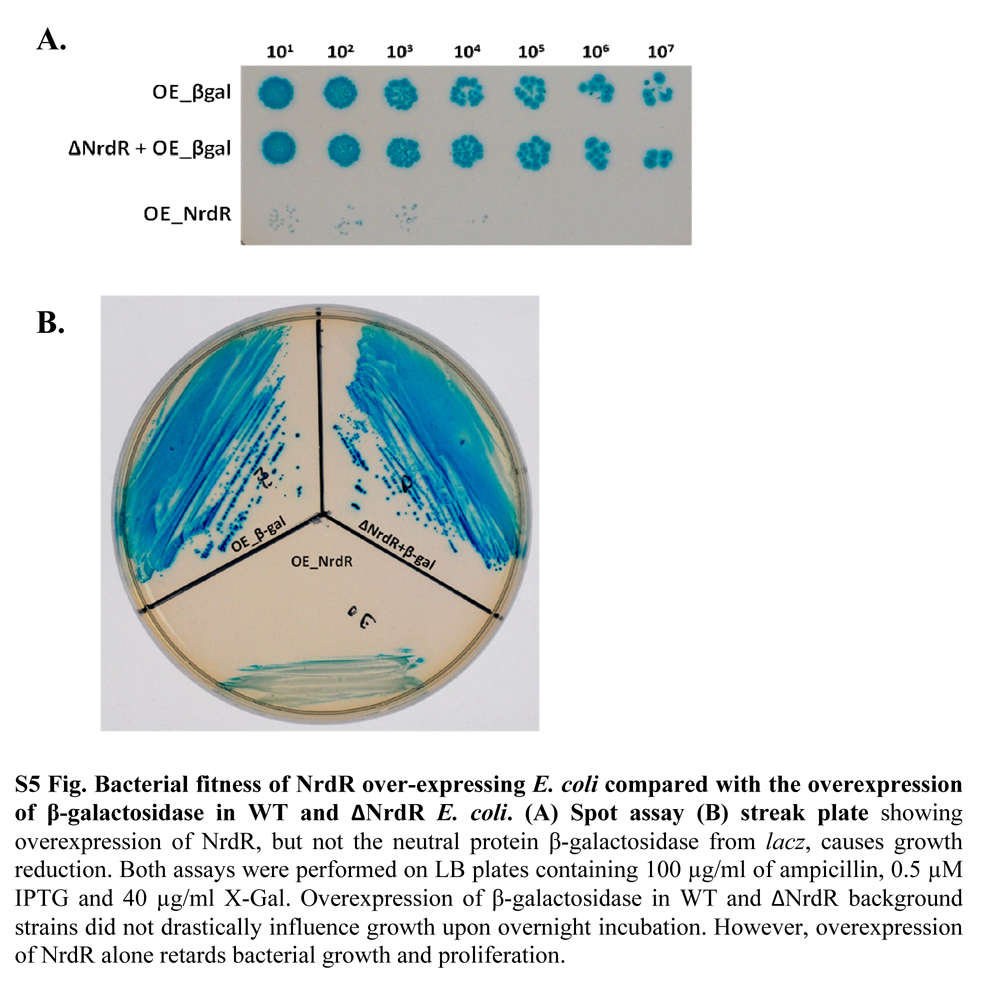

Supplement: S5 Fig — (A) Spot assay (B) streak plate showing overexpression of NrdR, but not the neutral protein β-galactosidase from lacZ, causes growth reduction. Both assays were performed on LB plates containing 100 μg/ml of ampicillin, 0.5 μM IPTG and 40 μg/ml X-Gal. Overexpression of β-galactosidase in WT and ΔNrdR background strains did not drastically influence growth upon overnight incubation. However, overexpression of NrdR alone retards bacterial growth and proliferation. (TIF) [file pone.0157165.s005.tif]

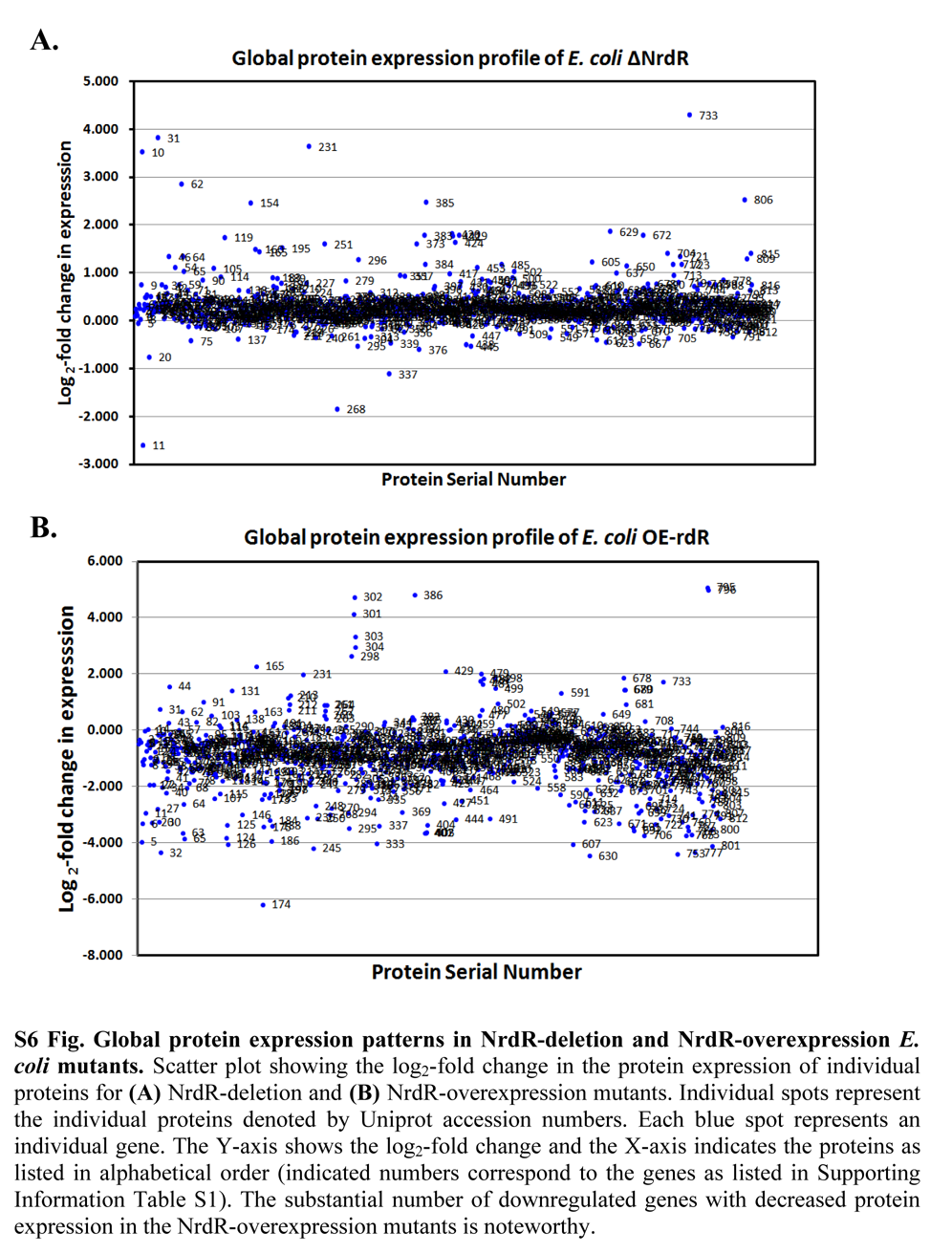

Supplement: S6 Fig — Scatter plot showing the log2-fold change in the protein expression of individual proteins for (A) NrdR-deletion and (B) NrdR-overexpression mutants. Individual spots represent the individual proteins denoted by Uniprot accession numbers. Each blue spot represents an individual gene. The Y-axis shows the log2-fold change and the X-axis indicates the proteins as listed in alphabetical order (indicated numbers correspond to the genes as listed in S1 Table). The substantial number of downregulated genes with decreased protein expression in the NrdR-overexpression mutants is noteworthy. (TIF) [file pone.0157165.s006.tif]

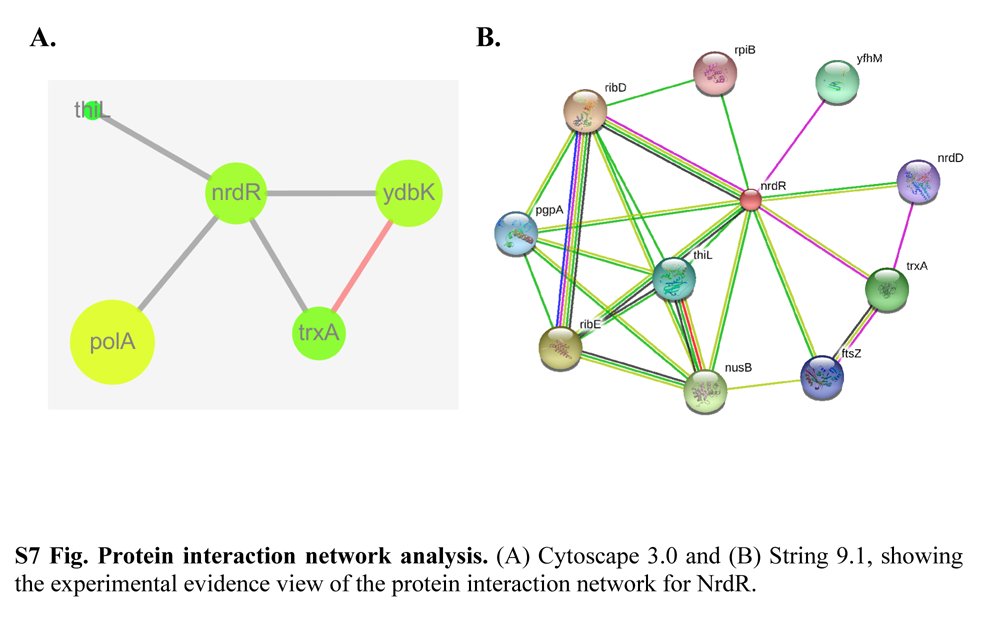

Supplement: S7 Fig — (A) Cytoscape 3.0 and (B) String 9.1, showing the experimental evidence view of the protein interaction network for NrdR. (TIF) [file pone.0157165.s007.tif]

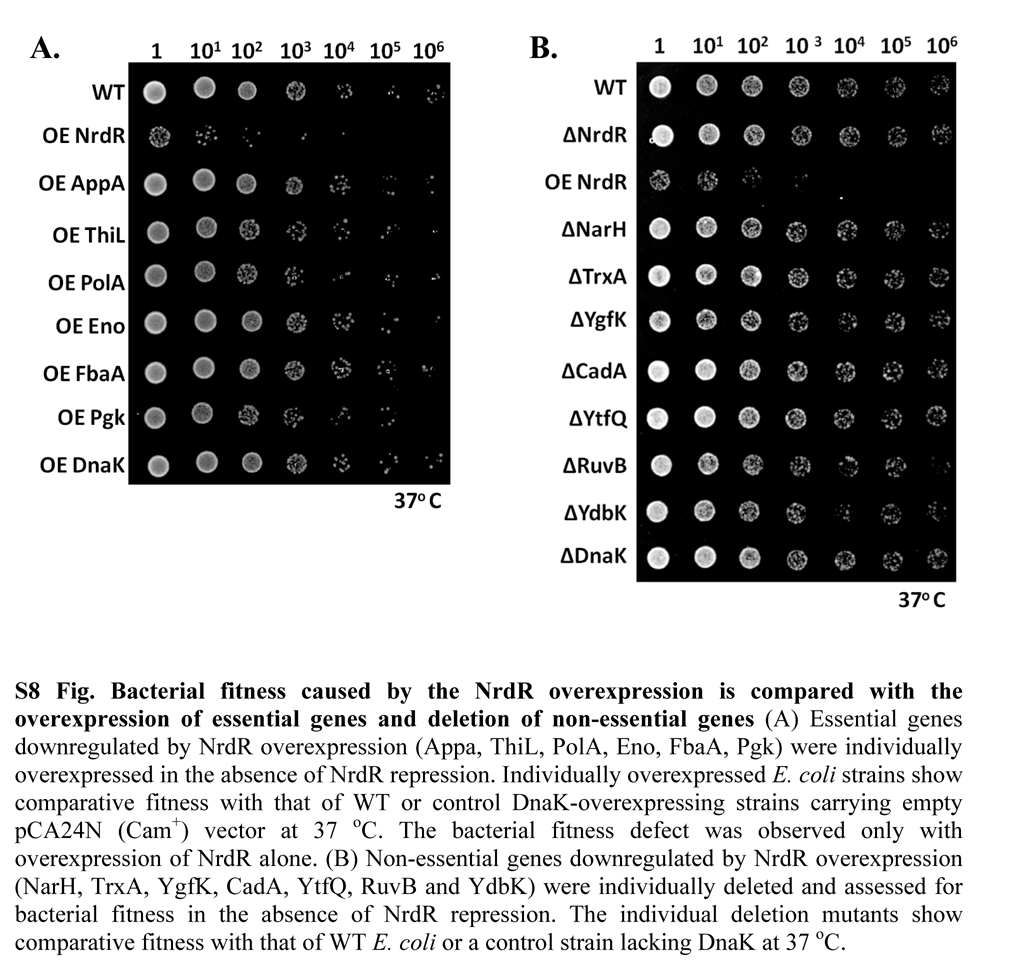

Supplement: S8 Fig — (A) Essential genes downregulated by NrdR overexpression (Appa, ThiL, PolA, Eno, FbaA, Pgk) were individually overexpressed in the absence of NrdR repression. Individually overexpressed E. coli strains show comparative fitness with that of WT or control DnaK-overexpressing strains carrying empty pCA24N (Cam+) vector at 37°C. The bacterial fitness defect was observed only with overexpression of NrdR alone. (B) Non-essential genes downregulated by NrdR overexpression (NarH, TrxA, YgfK, CadA, YtfQ, RuvB and YdbK) were individually deleted and assessed for bacterial fitness in the absence of NrdR repression. The individual deletion mutants show comparative fitness with that of WT E. coli or a control strain lacking DnaK at 37°C. (TIF) [file pone.0157165.s008.tif]

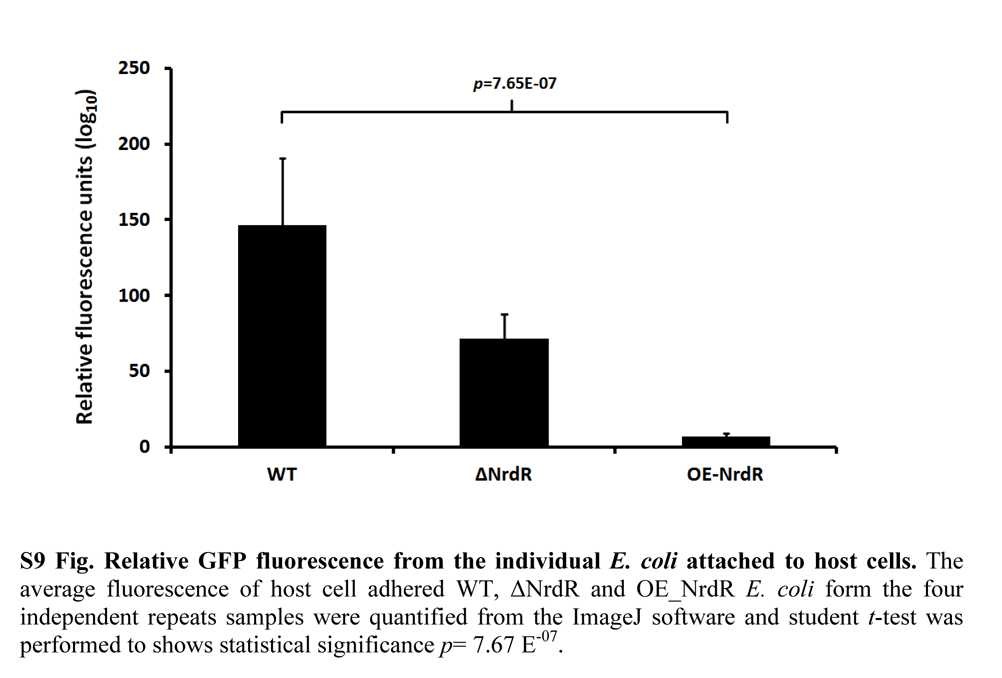

Supplement: S9 Fig — The average fluorescence of host cell adhered WT, ΔNrdR and OE_NrdR E. coli form the four independent repeats samples were quantified from the ImageJ software and student t-test was performed to shows statistical significance p = 7.67 E-07. (TIF) [file pone.0157165.s009.tif]
